# Caught while Dissolving: Revealing the Interfacial Solvation of the Mg^2+^ Ions on the MgO Surface

**DOI:** 10.1021/acsami.2c10005

**Published:** 2022-08-15

**Authors:** Francesco Tavani, Matteo Busato, Luca Braglia, Silvia Mauri, Piero Torelli, Paola D’Angelo

**Affiliations:** † Dipartimento di Chimica, Università di Roma “La Sapienza”, P.le A. Moro 5, 00185 Roma, Italy; ‡ CNR - Istituto Officina dei Materiali, TASC, I-34149 Trieste, Italy; § Dipartimento di Fisica, Università di Trieste, Via A. Valerio 2, 34127 Trieste, Italy

**Keywords:** XAS, NEXAFS, MCR analysis, MgO, metal oxide−water interface

## Abstract

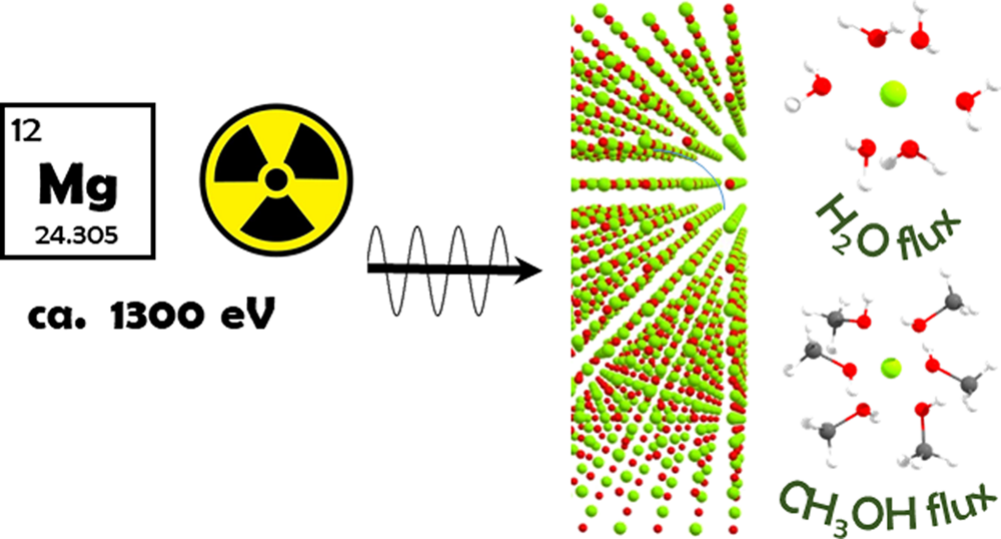

Interfaces between water and materials are ubiquitous and are crucial in materials sciences and in biology, where investigating the interaction of water with the surface under ambient conditions is key to shedding light on the main processes occurring at the interface. Magnesium oxide is a popular model system to study the metal oxide–water interface, where, for sufficient water loadings, theoretical models have suggested that reconstructed surfaces involving hydrated Mg^2+^ metal ions may be energetically favored. In this work, by combining experimental and theoretical surface-selective ambient pressure X-ray absorption spectroscopy with multivariate curve resolution and molecular dynamics, we evidence in real time the occurrence of Mg^2+^ solvation at the interphase between MgO and solvating media such as water and methanol (MeOH). Further, we show that the Mg^2+^ surface ions undergo a reversible solvation process, we prove the dissolution/redeposition of the Mg^2+^ ions belonging to the MgO surface, and we demonstrate the formation of octahedral [Mg(H_2_O)_6_]^2+^ and [Mg(MeOH)_6_]^2+^ intermediate solvated species. The unique surface, electronic, and structural sensitivity of the developed technique may be beneficial to access often elusive properties of low-Z metal ion intermediates involved in interfacial processes of chemical and biological interest.

## 1 Introduction

Achieving accurate chemical knowledge on the mechanisms of surface processes is of considerable interest both for fundamental understanding and for applications. Surfaces, in fact, provide unique platforms for the success of desirable reactive or nonreactive pathways, for instance by acting toward them as efficient energy-dissipating heat baths and by altering their symmetries significantly if compared to those in the gas or condensed phases.^1^ As a consequence, interfaces display uncanny properties that may greatly differ from those of the bulk, and innovative experimental and theoretical methods are required to disentangle the intricate mechanisms involved in surface science.

The metal oxide–water interface is of paramount importance in catalysis, materials science, biology, corrosion, geochemistry, and interstellar and atmospheric chemistry.^2−4^ Magnesium oxide has been frequently investigated as a model system, being one of the simplest oxides in terms of geometric and electronic structure. The interaction of a number of MgO surfaces with water^4−6^ has been studied both experimentally and theoretically at various temperatures and pressures in the range between high vacuum and ambient conditions, with the MgO(001) slab being one of the most popularly evaluated by researchers.^3,7−15^ While it is known that for low water coverage on the MgO(001) surface water forms a layer where 1/5 and 1/3 of the water molecules are dissociated at low (100–180 K) and higher (185–221 K) temperatures, respectively, for water coverages beyond a monolayer, the picture is less definite.^16^ In the latter case the standard model^7^ of a fully hydroxylated MgO(001) surface, where OH^–^ and H^+^ ions form by dissociated adsorption of one water molecule per MgO surface pair and coordinate the surface Mg^2+^ and oxygen ions, respectively, has been put into question. The extent of MgO(001) surface hydroxylation has not in fact been settled by the numerous experiments conducted at ambient water conditions,^5,16^ and recently it has been found that reconstructed surfaces, involving hydrated/hydroxylated Mg^2+^ ions above the MgO(001) surface, are more stable than the fully hydroxylated ones.^16^ It appears therefore natural to resort to surface-specific advanced experimental techniques to address the question of which is the prevalent Mg^2+^ species at the MgO–water interface in ambient pressure conditions. In principle, the molecular-level rationalization of the interactions established by the metal oxide surface with water requires (i) quantitative structural details, and (ii) information on the electronic states of the arising surface species.^17^ Among the cutting-edge experimental techniques that may simultaneously provide such information, X-ray absorption spectroscopy (XAS) set itself apart as an advanced tool that offers insights into the local structural and electronic environment of a selected photoabsorbing atom with an unrivaled degree of accuracy.^18−20^ However, to date, the use of XAS to probe the intermediate species formed at low-Z metal oxide surfaces when these interact with water and other organic solvents has been quite limited by the requirement of soft X-rays (∼400–2000 eV), that need tailored experimental setups.^17,21^ XAS in the hard X-ray regime has been widely employed for the investigation of the properties of 3d transition metals and operando XAS experiments with hard X-rays are routinely performed.^19^ On the contrary, the application of XAS in the soft X-ray regime (soft-XAS) to study the interfacial properties of metal ions has been severely hampered by the need of high vacuum conditions. Very recently, specific cells have been designed that allow soft-XAS to be carried out at atmospheric pressure under operando conditions,^22−25^ a technique referred to as ambient pressure near-edge X-ray fine structure spectroscopy (AP-NEXAFS). In this case soft-XAS is operated in total electron yield (TEY) detection mode which renders the technique surface sensitive due to the low electron escape depth which limits the thickness of the probed sample. Here, we use soft-XAS operando experiments in combination with state of the art chemometric and theoretical analyses to investigate the MgO surface upon interaction with water and methanol (MeOH). We found that Mg^2+^ ions are reversibly hydrated (solvated)/dehydrated (desolvated) at the interface, and we developed a novel experimental approach able to follow in real time the evolution of low-Z metal ion-based interfaces.

## 2 Experimental Methods

We provide a brief description of the experimental method, while the details and theoretical background concerning the data processing, multivariate curve resolution (MCR) analysis, density functional theory (DFT) cluster optimization, molecular dynamics (MD) simulations, and NEXAFS calculations are reported in the Supporting Information (SI). The experiments were carried out at the APE High Energy beamline at the Elettra Synchrotron radiation source (Basovizza, Italy). AP-NEXAFS operando measurements were enabled by the use of a specially designed reaction cell. The samples inside the reactor cell can be heated from room temperature to approximately 400 °C and can be exposed to a flux of different gases at a pressure of 1 bar. Figure S1a shows the 3D rendering of the operando NEXAFS reaction cell designed at the APE-HE beamline. On the top of the cell, a Si_3_N_4_ membrane is mounted (orange circle in Figure S1a), which separates the volume of the reactor cell at atmospheric pressure (labeled with **1** in Figure S1b) from the ultrahigh vacuum (UHV) environment of the beamline, while allowing the passage of the soft X-rays. The sample is positioned inside the reactor, normal to the incident X-rays (**2**). Two pipes (inlet and outlet) are connected to the reactor (**3**), allowing the circulation of gas inside the reactor (also during spectra acquisition). The heating of the sample is possible thanks to a ceramic heater installed below the sample, outside the reactor (**4**). The NEXAFS spectra are recorded in the TEY mode: two electrical contacts (one on the 100 nm thick Si_3_N_4_ membrane and one on the sample holder) allow one to polarize the membrane (positively to accelerate the electrons away from the sample) and measure the drain current of the sample through a picoammeter. The measurements were performed through the picoammeter, keeping the sample grounded and applying a positive bias voltage of 40 V to the membrane.

MgO was purchased from Sigma-Aldrich. The powder was fixed on a titanium sample holder and pressed in a pit located onto the holder. The cell was mounted in the UHV chamber coaxially with the X-ray beam. The MgO starting sample was pretreated at a temperature *T* = 250 °C in flowing He at 50 standard cubic centimeters per minute (SCCM), after which the working temperature was lowered to *T* = 50 °C. The experiments were performed collecting the Mg K-edge spectra in the energy range 1275–1355 eV at *T* = 50 °C and under flowing gas mixtures of 3% H_2_O/He or 17% MeOH/He, in both cases at 50 SCCM and 1 bar. Each operando AP-NEXAFS spectrum was recorded in approximately 5 min.

## 3 Results and Discussion

The newly developed AP-NEXAFS technique is a powerful method to unveil the structural properties and the processes occurring at the surface of a material during exposure to water or organic solvents, due to its surface sensitivity. In this work we applied this innovative experimental method, in combination with a state of the art theoretical approach to investigate the MgO–water and MgO–methanol interfaces at ambient pressure. Our experimental procedure was divided into four consecutive steps:

1. Initially, a clean MgO sample (pretreated in He at 250 °C to eliminate superficial impurities) was exposed to water vapor for 95 min using He as a carrier gas at a working temperature of 50 °C.
2. In the second step, the water flux was interrupted and the temperature was increased up to 250 °C with a 3.3 °C per minute rate while exposing the sample to He.
3. To investigate the superficial interaction of MgO with an organic solvent, the same sample was then exposed to methanol vapor (with He acting as a carrier gas) at a temperature of 50 °C for 85 min.
4. Finally, the methanol flux was interrupted and the temperature of the system was increased up to 250 °C with a 6.6 °C per minute rate while exposing the sample to an inert atmosphere.

Operando AP-NEXAFS spectra were collected at the Mg K-edge every 5 min throughout the duration of the entire experiment. Tables S1 and S2 of the SI list the temperatures at which all the spectra were recorded during the exposure of MgO to water and methanol vapors, respectively. The measured XAS data were then subjected to a mathematical decomposition procedure using a strategy belonging to the MCR family. This method allows the rationalization of operando spectroscopic data sets,^26−30^ leading to the retrieval of the spectral and concentration profiles of the key Mg^2+^ species contributing to the experimental signal. Finally, the extracted Mg K-edge XAS spectra were quantitatively analyzed through ab initio DFT NEXAFS calculations and MD simulations.

**Figure 1:**
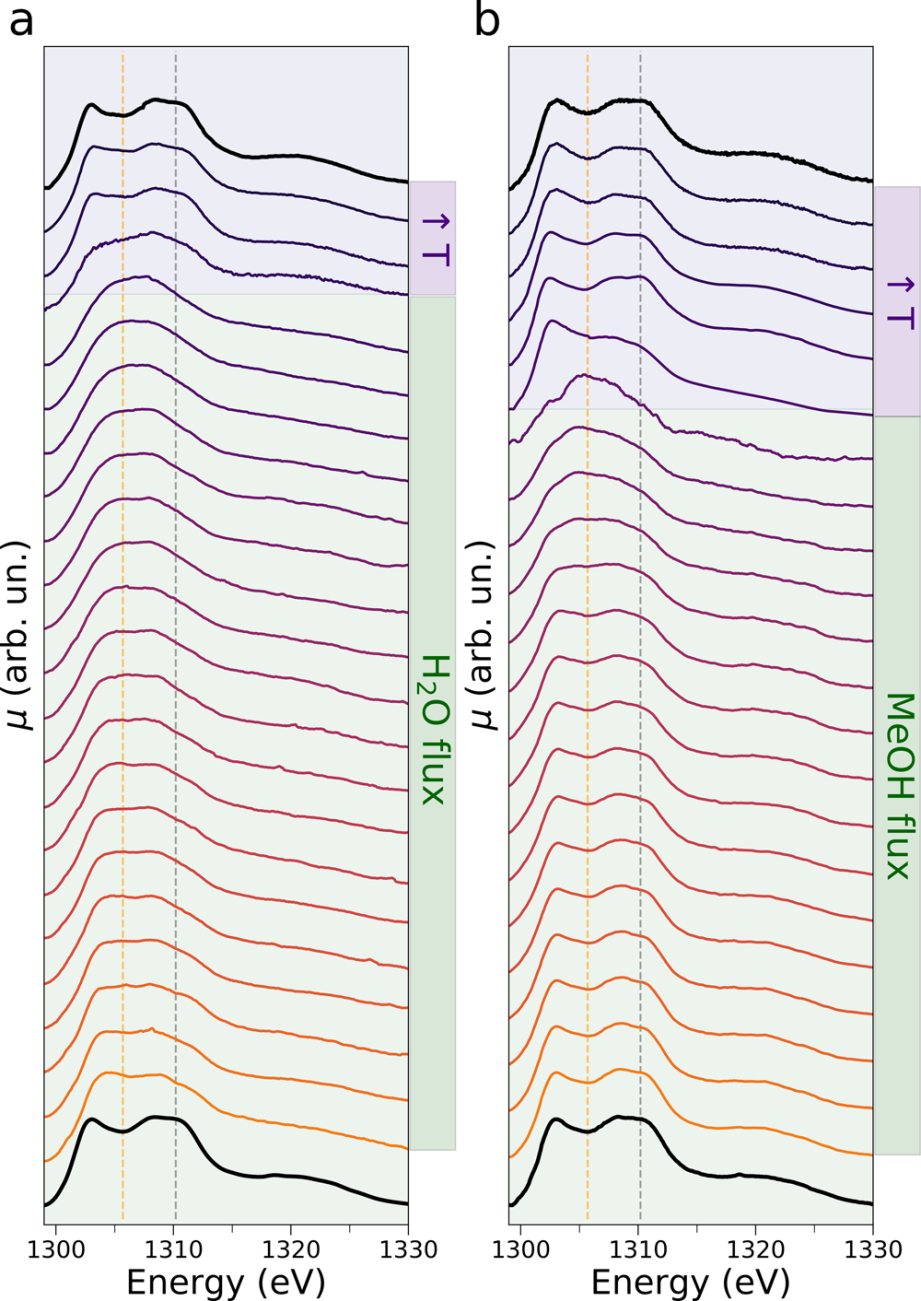
Evolution of the operando Mg K-edge AP-NEXAFS spectra upon MgO exposure to water (a) and methanol (b). Constant energy cuts are drawn at 1305.7 eV (yellow dotted lines) and at 1310.2 eV (gray dotted lines). The spectral scans recorded during the flux of water and methanol at 50 °C and during the subsequent flux interruption and temperature increase up to 250 °C are highlighted using green and purple backgrounds, respectively. In both panels, the AP-NEXAFS spectra recorded before the surface exposure to the given flux and at the temperature of 250 °C after flux interruption are evidenced in bold black lines.

Figures 1a and S3a present in two and three dimensions, respectively, the operando Mg K-edge AP-NEXAFS spectra recorded on the MgO sample during its exposure to water vapor at 50 °C (green background, step 1) and after flux interruption and temperature increase up to 250 °C (purple background, step 2). The first and last XAS spectra are highlighted by dark black lines (Figure 1a) and were measured, respectively, on the pristine MgO sample prior to water exposure and at the final temperature of 250 °C after water flux interruption.

**Figure 2:**
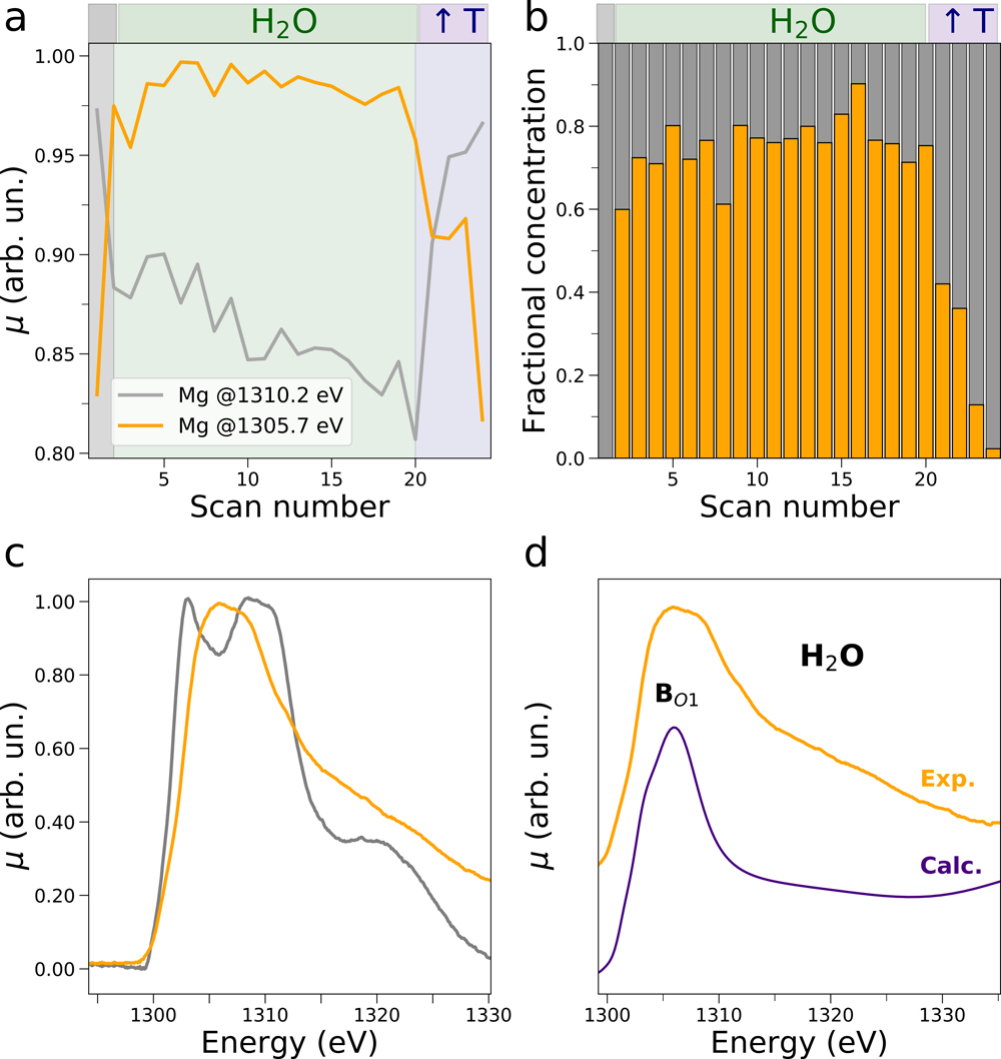
(a) Intensity variation at 1305.7 eV (full yellow line) and at 1310.2 eV (full gray line) of the operando Mg K-edge XAS spectra measured upon MgO exposure to water vapor. The intensities of the starting XAS spectrum, of the XAS spectra recorded during the water flux at 50 °C, and of those measured during the water flux interruption and contemporary temperature increase are highlighted by black, green, and purple backgrounds, respectively. (b, c) Results of the decomposition of the Mg K-edge spectra. Extracted concentration profiles and Mg K-edge NEXAFS spectra (panels b and c, respectively). (d) Comparison between the NEXAFS spectrum of the Mg^2+^ intermediate species arising upon exposure of the MgO surface to water and the theoretical average Mg K-edge NEXAFS spectrum resulting from 500 MD snapshots of the fully hydrated Mg^2+^ ion (full indigo line).

Looking at Figure 1a, one may note that during the exposure of the pristine MgO surface to water vapor, there is an appreciable spectral variation. In particular, the feature at 1310.2 eV in the initial Mg K-edge spectrum of the pristine MgO surface decreases in intensity while a feature located at 1305.7 eV appears, as evidenced by the constant energy cuts at the same energy values. The intensity time evolution of these spectral features is displayed in Figure 2a. Conversely, once the water flux is interrupted and the temperature is progressively increased to 250 °C (Figure 1a, purple background), the intensities of the features located at 1310.2 eV and at 1305.7 eV rapidly increase and decrease, respectively, to their initial values and the overall spectral appearance of the starting MgO spectrum is fully recovered. Notably, the first and last AP-NEXAFS experimental spectra are nearly superimposable, thereby strongly suggesting the complete reversibility of the temperature-assisted Mg^2+^–water interaction at the MgO surface. One may also observe in Figure 2a that, upon water vapor exposure, the decrease in intensity of the characteristic feature located at 1310.2 eV nearly mirrors the increase of intensity of the one at 1305.7 eV. This finding qualitatively suggests that two interconverting Mg^2+^ active species contribute to the measured AP-NEXAFS data. To obtain quantitative information on the number of pure chemical species contributing to the experimental XAS spectra, the percentage residual error committed in reconstructing the data set with an increasing number of components was evaluated, as shown in Figure S4a (refer to the SI for additional details). One may note that the percentual error committed in employing a number of principal components greater than two to reproduce the data set decreases very slowly, and that the percentual error committed in employing two components to reproduce the AP-NEXAFS data is ca. 3%. These evidence suggest the presence of two main components in the operando Mg K-edge NEXAFS spectra measured upon exposure of the MgO sample to water vapor.

To gain mechanistic and structural insights into the nature of the interaction established by the surface Mg^2+^ ions with the fluxed water, the experimental AP-NEXAFS data were analyzed using an MCR transition matrix (TM)-based decomposition approach employing a number of significant components equal to 2, a method fully discussed in the SI. Figures 2b,c show the extracted concentration and spectral profiles, where the first extracted spectral component (Figure 2c, gray curve) was constrained to coincide with the AP-NEXAFS spectrum of the pristine MgO surface (Figure 1a, initial dark black spectrum). The MgO material possesses good thermal stability above 600 °C^31^ and has been thoroughly investigated by previous solid-state XAS studies.^32−35^

The extracted MgO XAS spectrum is in excellent agreement with previous MgO K-edge XAS measurements exhibiting a transition at 1303.0 eV and two experimentally unresolved features at 1308.0 eV and at 1310.7 eV, together with a broad shoulder at 1320.6 eV.^32,35^ The second XAS component shown in Figure 2c (yellow curve) presents a single broad feature centered at ∼1305.7 eV and is assigned to the arising Mg^2+^ intermediate species due to the interaction of the Mg^2+^ surface ions with water. In fact, as shown in Figure 2b, the fractional concentration of the latter component increases rapidly during water flux, reaching values close to 75%, while the fractional concentration of MgO is largely predominant once the temperature reaches 250 °C, when water is expected to be fully desorbed from the surface. Further, we have observed an approximate 5-fold decrease in spectral intensity of the raw non-normalized XAS spectra during exposure of the MgO surface to water vapor, and consequently we may estimate the interaction between water and the interfacial Mg^2+^ ions to extend to the first few nanometers below the surface. Interestingly, the XAS spectrum of the Mg^2+^ intermediate species closely resembles the Mg K-edge XAS spectrum previously reported for [MgCl_2_(H_2_O)_6_] in aqueous solution and qualitatively assigned to that of a fully dissolved octahedral hexaquo Mg^2+^ ion.^36^ The presented evidence suggests that the surface Mg^2+^ ions do interact with the incoming water molecules, as evidenced by the different electronic features of the two intermediate extracted AP-NEXAFS components, and that a surface Mg^2+^ dissolution process occurs.

To test these hypotheses and to uncover the structural and electronic properties of the Mg^2+^–water intermediate, an ab initio DFT-based NEXAFS theoretical analysis was carried out,^37−40^ with the support of MD simulations. First, to verify the validity of the implemented framework, the theoretical NEXAFS spectrum of MgO was calculated starting from the available rock-salt crystal structure (space group *Fm*3̅*m*, and lattice parameter of 4.21 Å).^41^Figure S5 reports the theoretical spectrum calculated for the MgO crystal (full black line), along with the calculated Mg- and O-density of electronic p states (DOS), compared to the experimental MgO spectrum obtained from the multivariate analysis (full gray line). One may note that both Mg p- and O p-states contribute through hybridization to the four main calculated features **A**_**1**_, **A**_**2**_, **A**_**3**_, and **A**_**4**_, whose energy positions and relative intensities are in excellent agreement with those of the features in the experimental MgO spectrum. Having established the reliability of our theoretical approach, to explore its sensitivity to the local structural properties of the Mg^2+^ ion, we performed theoretical NEXAFS simulations on DFT-optimized [Mg(H_2_O)_*n*_]^2+^ molecular clusters (with *n* = 4, 6),^42−44^ where the oxygen atoms coordinate the central metal cation in a tetrahedral and octahedral geometry, respectively. Previous investigations have reported structural features on hydrated Mg^2+^ both from experiments and from computer theoretical simulations, thereby providing reliable findings on which to benchmark our approach. In aqueous solution, an octahedral hexaquo Mg^2+^ has been evidenced through Raman spectroscopy,^45^ proton NMR,^46^ and an X-ray diffraction experiment conducted by difference methods,^47^ and results were confirmed by Monte Carlo,^48^ MD, and ab initio quantum mechanical/molecular mechanical (QM/MM) MD simulations.^49−51^ The average Mg–O bond distances of our DFT octahedral [Mg(H_2_O)_6_]^2+^ structure was found to be equal to 2.10 Å, as listed in Table S3, and is in very good agreement with the previously reported bond length of 2.110 Å.^52^ The Mg K-edge theoretical spectra calculated for the [Mg(H_2_O)_4_]^2+^ and [Mg(H_2_O)_6_]^2+^ complexes are shown in Figure S6a and S6b, respectively (full black lines), along with the associated Mg p- and O p- DOS. The overall shape of the two spectra is very different, and this shows how sensitive this technique is to the coordination of the Mg photoabsorber. In particular, the theoretical convoluted NEXAFS spectrum of the [Mg(H_2_O)_4_]^2+^ complex exhibits two clearly distinguishable transitions, **B**_*T***1**_ and **B**_*T***2**_, while that of the [Mg(H_2_O)_6_]^2+^ species possesses a single main transition (**B**_*O***1**_) together with a less pronounced high-energy shoulder (**B**_*O***2**_). The latter spectrum bears significant resemblance to the MCR-extracted XAS spectrum of the Mg^2+^ species interacting with water (Figure 2c, full yellow line) and with previous Mg K-edge spectra attributed to the fully hydrated Mg^2+^ ion in aqueous solution, both of which present a single broad main transition.^36^

**Figure 3:**
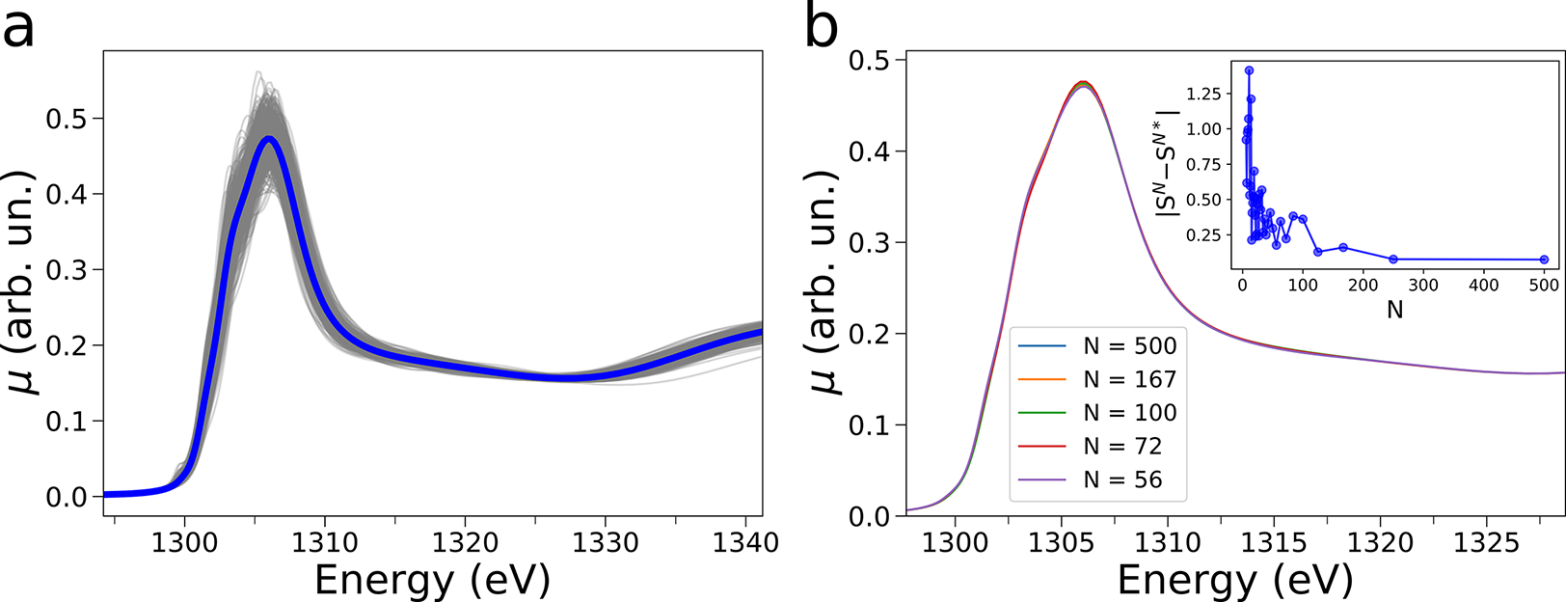
Theoretical NEXAFS spectra (gray full lines) calculated from 500 MD snapshots of the Mg^2+^ ion in water at 50 °C and converged NEXAFS average (blue full line) of the 500 spectra (a) along with a selection of average NEXAFS spectra calculated with a variable number (*N*) of spectra (b). The associated evolutions of the total absolute differences between averages of spectra computed with increasing *N* values are shown in the insets of panel b. *S*^*N*^ and *S*^N*^ are the averages of *N* and N* spectra, respectively, with N* immediately preceding the given value of *N* in the evaluated sequence (e.g., N* = 250 if *N* = 500).

For poorly ordered systems as in the case of the Mg^2+^ hydrated species formed on the MgO surface, the NEXAFS signal originates from the average over all the possible configurations adopted by water molecules around the ion, and a single cluster cannot be used to correctly reproduce the NEXAFS spectrum.^53−55^ To overcome this problem and to properly account for thermal and structural fluctuations occurring at 50 °C, we performed a quantitative analysis of the NEXAFS spectra starting from the microscopic description of the system derived from the MD simulations. This combined method is very powerful, as disorder effects due to the dynamic distortions of the coordination shells are properly included in the calculation of the NEXAFS theoretical spectrum. In particular, classical MD simulations of the Mg^2+^ ion in water were performed and details are discussed in the SI. In water an octahedral geometry with coordination number (CN) of 6 and a Mg–O bond length of 2.10 Å were obtained for the first hydration shell molecules, as evidenced by the Mg–O radial distibution function and its corresponding running integration number (see Figure S7a). Next, an averaged NEXAFS theoretical spectrum has been calculated starting from the structural configurations obtained from 500 MD snapshots, and the resulting spectra are shown in Figure 3a. In all cases, the NEXAFS spectra calculated from each MD snapshot present detectable differences in all the energy range, showing the sensitivity of NEXAFS to geometrical changes and fluctuations of the Mg^2+^ hydration clusters and the importance of making a proper sampling of the configurational space.

Figure 3b presents the NEXAFS theoretical spectral averages for the dissolved Mg^2+^ ion system computed with a variable number (*N*) of spectra, where *N* belongs to a monotonely increasing sequence. One may note that the theoretical averaged NEXAFS present very small differences for *N* > 50 (see Figure 3b) and that they are well-converged for *N* = 500 (inset of Figure 3b). The NEXAFS converged average spectrum is compared in Figure 2d to the experimental NEXAFS spectrum of the intermediate Mg^2+^ species arising at the MgO surface upon its exposure to water vapor at 50 °C. The agreement between the theoretical and experimental curves is quite good, and the former spectrum shows a certain degree of the experimentally observed configurational broadening if compared to the NEXAFS calculation performed on the DFT-optimized [Mg(H_2_O)_6_]^2+^ cluster (Figure S6b, full black line). These findings strongly corroborate the picture that within our experimental conditions Mg^2+^ ions are partially released from the MgO surface and are coordinated at the interface in an octahedral geometry by water molecules, as described by our theoretical framework.

**Figure 4:**
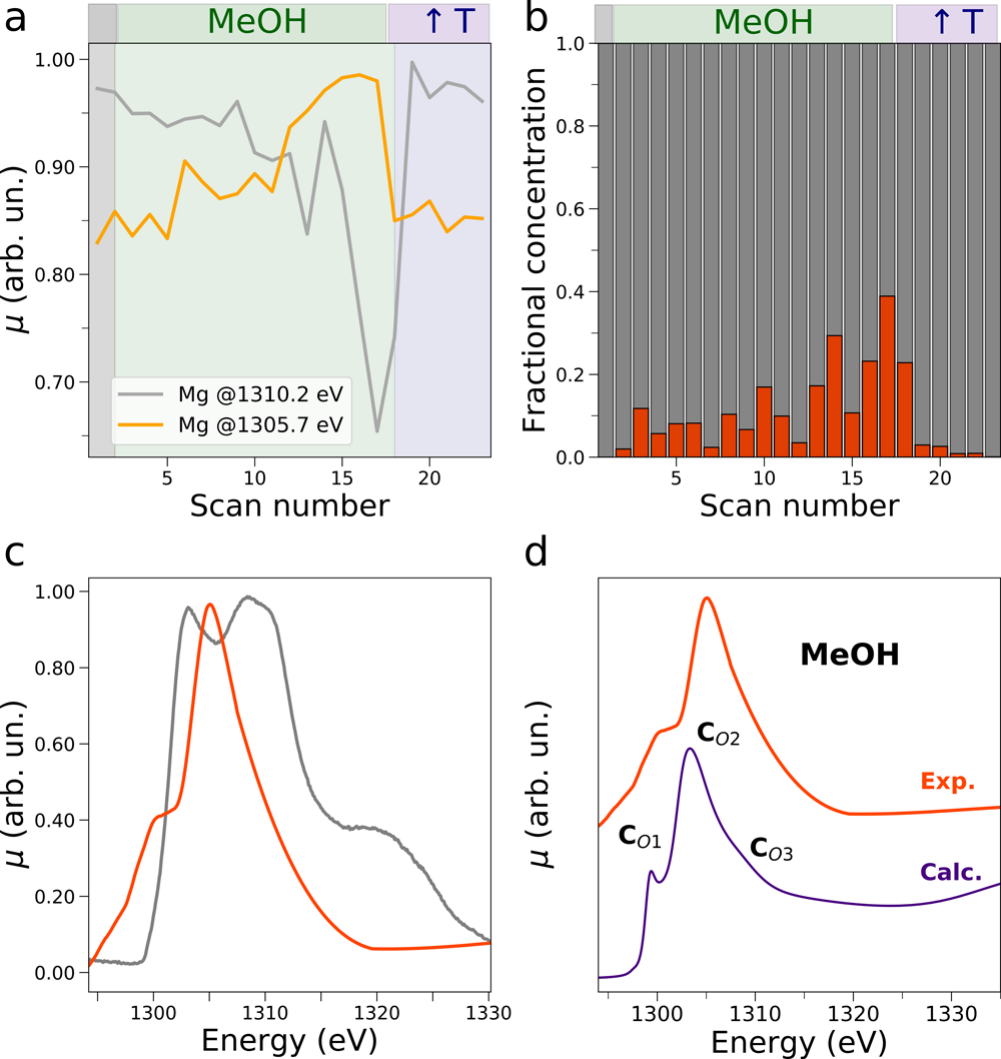
(a) Intensity variation at 1305.7 eV (full yellow line) and at 1310.2 eV (full gray line) of the operando Mg K-edge XAS spectra measured upon MgO exposure to methanol vapor. The intensities of the starting XAS spectrum, of the XAS spectra recorded during the methanol flux at 50 °C, and of those measured during the methanol flux interruption and contemporary temperature increase are highlighted by black, green, and purple backgrounds, respectively. (b, c) Results of the decomposition of the Mg K-edge spectroscopic data. Extracted concentration and Mg K-edge NEXAFS spectral profiles (panels b and c, respectively). (d) Comparison between the MCR-extracted AP-NEXAFS spectral component of the Mg^2+^ intermediate species arising upon exposure of the MgO surface to methanol and the theoretical average Mg K-edge NEXAFS spectrum resulting from 500 MD snapshots of the Mg^2+^ ion fully solvated by methanol molecules (full indigo line).

This hypothesis is further supported by the subsequent operando experiment performed by exposing the same MgO sample to methanol vapor at 50 °C (step 3). Figures 1b and S3b show the operando Mg K-edge AP-NEXAFS spectra measured upon MgO exposure to methanol in 2D and 3D, respectively. Also in this experiment an appreciable NEXAFS spectral change occurs during methanol flux as evidenced by the decrease in intensity at 1310.2 eV and the corresponding increase in intensity at 1305.7 eV (see Figure 4b) and by the appearance of a low-energy transition at 1300.2 eV prior to flux interruption and temperature increase. It is important to notice that the first and last experimental spectra coincide with that of pristine MgO, suggesting that the interaction between the surface and methanol vapor is a reversible one and that the NEXAFS spectra obtained under the methanol flux are quite different from those obtained when fluxing water. Also in this case the NEXAFS signals were subjected to a MCR analysis, using a number of active components equal to two because (i) the percentual error committed by reproducing the data set using two principal components is inferior to 4% (Figure S4b), and (ii) the intensity time decay measured at 1310.2 eV closely mirrors the increase of that at 1305.7 eV, as shown in Figure 4a. Figure 4 parts b and c present, respectively, the concentration profiles and XAS spectra extracted from the matricial decomposition. Aside from the XAS spectrum of MgO, a second AP-NEXAFS spectrum contributes to the measured data (Figure 4c, full red line) and is assigned to an intermediate species arising from the interaction between Mg^2+^ surface ions and methanol. The XAS spectrum of this intermediate species presents a low-energy shoulder at 1300.2 eV together with an asymmetric main transition at 1305.1 eV, while its fractional concentration slowly increases upon methanol surface exposure never exceeding a value of 40% (Figure 4b, red histograms). It is interesting to outline that a smaller fraction of Mg^2+^ ions are fully solvated by methanol molecules as compared to the number of surface ions that are hydrated, and this is in agreement with the lower solubility of MgO in the former solvent. Similarly to the experiment involving water, also here the methanol molecules desorb from the MgO surface once the working temperature is increased to 250 °C and the fractional concentration of the MgO related component reaches 100% (Figure 4b, gray histograms). Note that also in this case the general intensity of the non-normalized XAS spectra decreases ca. 5-fold during exposure of the MgO surface to methanol vapor, thereby suggesting that the interaction between methanol and the Mg^2+^ ions extends to the first few nanometers below the surface. To determine the structural properties of the Mg^2+^–methanol interfacial intermediate, we calculated theoretical NEXAFS spectra on DFT-optimized [Mg(MeOH)_4_]^2+^ and [Mg(MeOH)_6_]^2+^ tetrahedral and octahedral clusters, which are shown in Figure S8a and S8b, respectively, and both present three main groups of peaks. In the case of the [Mg(MeOH)_4_]^2+^ species, the lowest energy peak identified as **C**_*T***1**_ is the most intense feature, while in the spectrum of [Mg(MeOH)_6_]^2+^ the feature located at intermediate energies, **C**_*O***2**_, is the most intense one, followed in relative intensity by the low-energy edge shoulder **C**_*O***1**_ and the highest energy peak **C**_*O***3**_. The origin of this difference in the relative feature intensity may be explained by analyzing the theoretical DOS evaluated for the two differently coordinated Mg^2+^–methanol clusters. In fact, in the case of the [Mg(MeOH)_4_]^2+^ complex, the Mg, O, and C p-DOS significantly overlap in the low-energy region of the NEXAFS spectrum, enabling a pronounced hybridization and an intense **C**_*T***1**_ transition (see Figure S8a), while for the [Mg(MeOH)_6_]^2+^ adduct, the octahedral environment leads to a decrease in intensity and hybridization of the Mg, O, and C p-DOS in the low-energy shoulder region (see Figure S8b), depleting the intensity of the feature **C**_*O***1**_. A visual comparison of the NEXAFS spectrum of [Mg(MeOH)_6_]^2+^ shown in Figure S8b with the AP-NEXAFS MCR-extracted spectrum associated with the Mg^2+^ species formed upon interaction of the MgO surface with methanol (Figure 4c, full red line) evidences that the theoretical and experimental curves show a strong degree of similarity. These results highlight the sensitivity of our theoretical method to the change in coordination number around the metal cation and strongly support the hypothesis that in our experimental conditions the Mg^2+^ ions at the MgO surface may indeed be coordinated in an octahedral geometry by methanol molecules. To further test this view, MD simulations were also performed for the Mg^2+^ ion in methanol at a temperature of 50 °C, obtaining a CN of 6 and a maximum in the Mg–O bond distribution of 2.12 Å for the first-shell molecules, as shown in Figure S7b, a distance that is in perfect agreement with the average 2.12 Å one in the DFT-optimized [Mg(MeOH)_6_]^2+^ cluster (Table S3). These results are well in line with previous investigations where X-ray diffraction,^56^ NMR,^57^ and ab initio MD^58^ studies have indicated that the Mg^2+^ ion solvate shells are composed of six methanol molecules, with a likely average octahedral arrangement of OH groups around the metal cation. The theoretical NEXAFS Mg K-edge spectrum of the Mg^2+^ ion in methanol was then calculated as a converged average from 500 MD snapshots (see Figure S9 for the individual MD-extracted XAS spectra and convergence details) and is compared to the experimental XAS signal in Figure 4d. As one may note from Figure 4d, the three main features located at 1300.2 eV, at 1305.1 eV, and at 1310.5 eV present in the experimental curve are reproduced well by the theoretical NEXAFS spectrum (Figure 4d, full indigo line).

**Figure 5:**
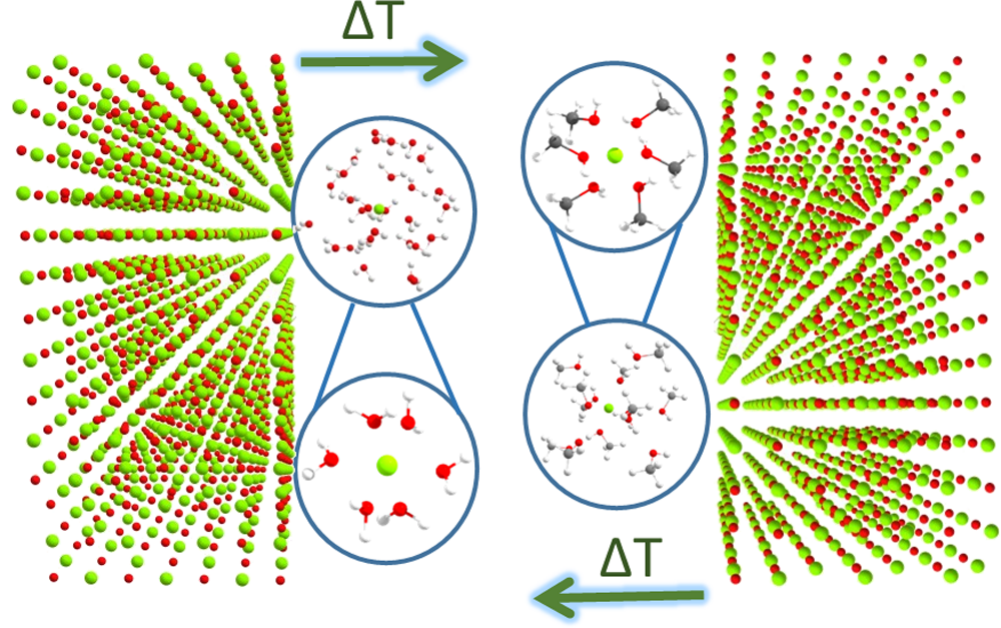
Pictorial representation of the main findings presented in this work, where it is evidenced that at 50 °C, a temperature-favored reversible hydration/solvation of Mg^2+^ ions occurs at the MgO surface, upon its sequential exposure to water (left) and methanol (right).

The findings reported herein may be summarized as shown in Figure 5. Within our experimental conditions, for high water and methanol coverages, Mg^2+^ ions are released at the MgO surface and fully solvated by water and methanol at 50 °C with temperature expected to play an important role in favoring ion mobility, release, and dissolution. The free Mg^2+^ ions are expected to be hydrated/solvated at the MgO surface in octahedral coordination geometries. Conversely, the generated surface O^2–^ ions are expected to transform into OH^–^ groups, as proposed by previous work,^16^ and to possibly yield a relatively smaller fraction of structures with partially hydroxylated Mg^2+^ ions above the surface.^16^ Given the very similar scattering properties of the water/OH^–^ species and low sensitivity of the XAS technique in distinguishing between water and OH^–^ ligands, one cannot exclude the presence of a small percentage of Mg^2+^ surface complexes where the water molecules in the first and outer coordination spheres are partially substituted by OH^–^ groups. Further, one cannot exclude the presence of a small fraction of Mg^2+^ ions possessing first and second shell structures affected by the structural modifications induced by the MgO surface to the first few adsorbed water/methanol layers. However, as evidenced by the presented statistical analyses the total contribution of all the differently hydrated/solvated Mg^2+^ ions is not expected to exceed 4%.

## 4 Conclusions

In this work, the suitability of a combined advanced MCR and ab initio DFT- and MD-assisted analysis of operando AP-NEXAFS data is demonstrated for the first time in order to access through X-rays and quantitatively describe the reversible hydration (solvation)/dehydration (desolvation) of a low-Z number metal oxide surface, investigating Mg^2+^ as a case study. It is shown that upon controlled exposure of MgO to water and methanol vapors at 50 °C and ambient pressure, surface Mg^2+^ ions form [Mg(H_2_O)_6_]^2+^ and [Mg(MeOH)_6_]^2+^ octahedral complexes, respectively, in detectable surface concentrations. These results provide a first direct experimental confirmation of previous theoretical findings which have suggested that at the MgO–water interface, reconstructed surfaces involving hydrated Mg^2+^ ions are energetically favored if compared to fully hydroxylated ones^16^ and extend the same picture to the MgO–methanol interface. The sensitivity of AP-NEXAFS to the structural and electronic evolution of the Mg^2+^ species and that of the MCR technique in uncovering the presence of labile surface intermediates are fruitfully exploited to gain quantitative information on a prototypical metal oxide–water/methanol interface. As a result, we expect this work to pave the way for the investigation of interfaces between water (and other organic solvating media) and metal ion-based solid systems through combined experimental and theoretical efforts rooted in soft-XAS.

## Supporting Information Available

Experimental details, data processing, MCR decomposition of the NEXAFS spectra, details on MD simulations and DFT calculations, 3D rendering of the employed reaction cell, theoretical NEXAFS calculations of DFT-optimized structures, MD Mg–O radial distribution functions of the Mg^2+^ ion in water and methanol, and convergence of the NEXAFS theoretical spectra calculated from MD snapshots of the Mg^2+^ ion in methanol (PDF)

## References

[ref1] Groß A. Reactions at Surfaces studied by Ab Initio Dynamics Calculations. Surf. Sci. Rep. 1998, 32, 291–340. 10.1016/S0167-5729(98)00008-9.

[ref2] Brown G. E.; Henrich V. E.; Casey W. H.; Clark D. L.; Eggleston C.; Felmy A.; Goodman D. W.; Grätzel M.; Maciel G.; McCarthy M. I.; Nealson K. H.; Sverjensky D. A.; Toney M. F.; Zachara J. M. Metal Oxide Surfaces and Their Interactions with Aqueous Solutions and Microbial Organisms. Chem. Rev. 1999, 99, 77–174. 10.1021/cr980011z. 11848981

[ref3] Carrasco E.; Brown M. A.; Sterrer M.; Freund H.-J.; Kwapien K.; Sierka M.; Sauer J. Thickness-Dependent Hydroxylation of MgO(001) Thin Films. J. Phys. Chem. C 2010, 114, 18207–18214. 10.1021/jp105294e.

[ref4] Verdaguer A.; Sacha G. M.; Bluhm H.; Salmeron M. Molecular Structure of Water at Interfaces: Wetting at the Nanometer Scale. Chem. Rev. 2006, 106, 1478–1510. 10.1021/cr040376l. 16608188

[ref5] Ewing G. E. Ambient Thin Film Water on Insulator Surfaces. Chem. Rev. 2006, 106, 1511–1526. 10.1021/cr040369x. 16608189

[ref6] Woodruff D. P. Quantitative Structural Studies of Corundum and Rocksalt Oxide Surfaces. Chem. Rev. 2013, 113, 3863–3886. 10.1021/cr3002998. 23294098

[ref7] Anderson P. J.; Horlock R. F.; Oliver J. F. Interaction of Water with the Magnesium Oxide Surface. Trans. Faraday Soc. 1965, 61, 2754–2762. 10.1039/tf9656102754.

[ref8] Imad-Uddin Ahmed S.; Perry S. S.; El-Bjeirami O. Desorption and Reaction of Water on MgO(100) Studied as a Function of Surface Preparation. J. Phys. Chem. B 2000, 104, 3343–3348. 10.1021/jp9934275.

[ref9] Johnson M. A.; Stefanovich E. V.; Truong T. N.; Günster J.; Goodman D. W. Dissociation of Water at the MgO(100)–Water Interface: Comparison of Theory with Experiment. J. Phys. Chem. B 1999, 103, 3391–3398. 10.1021/jp983729r.

[ref10] Giordano L.; Goniakowski J.; Suzanne J. Partial Dissociation of Water Molecules in the (3 × 2) Water Monolayer Deposited on the MgO (100) Surface. Phys. Rev. Lett. 1998, 81, 1271–1273. 10.1103/PhysRevLett.81.1271.

[ref11] Odelius M. Mixed Molecular and Dissociative Water Adsorption on MgO [100]. Phys. Rev. Lett. 1999, 82, 3919–3922. 10.1103/PhysRevLett.82.3919.

[ref12] Delle Site L.; Alavi A.; Lynden-Bell R. M. The Structure and Spectroscopy of Monolayers of Water on MgO: an Ab Initio Study. J. Chem. Phys. 2000, 113, 3344–3350. 10.1063/1.1287276.

[ref13] Jug K.; Heidberg B.; Bredow T. Cyclic Cluster Study of Water Adsorption Structures on the MgO(100) Surface. Surf. Sci. 2007, 601, 1529–1535. 10.1016/j.susc.2006.12.092.

[ref14] Lynden-Bell R.; Delle Site L.; Alavi A. Structures of Adsorbed Water Layers on MgO: an Ab Initio Study. Surf. Sci. 2002, 496, L1–L6. 10.1016/S0039-6028(01)01669-7.

[ref15] Jug K.; Heidberg B.; Bredow T. Molecular Dynamics Study of Water Adsorption Structures on the MgO(100) Surface. J. Phys. Chem. C 2007, 111, 6846–6851. 10.1021/jp067651n.

[ref16] Ončák M.; Włlodarczyk R.; Sauer J. Water on the MgO(001) Surface: Surface Reconstruction and Ion Solvation. J. Phys. Chem. Lett. 2015, 6, 2310–2314. 10.1021/acs.jpclett.5b00885. 26266610

[ref17] Akabayov B.; Doonan C. J.; Pickering I. J.; George G. N.; Sagi I. Using Softer X-ray Absorption Spectroscopy to Probe Biological Systems. J. Synchr. Rad. 2005, 12, 392–401. 10.1107/S0909049505010150. 15968114

[ref18] Capocasa G.; Sessa F.; Tavani F.; Monte M.; Olivo G.; Pascarelli S.; Lanzalunga O.; Di Stefano S.; D’Angelo P. Coupled X-ray Absorption/UV-vis Monitoring of Fast Oxidation Reactions Involving a Nonheme Iron-Oxo Complex. J. Am. Chem. Soc. 2019, 141, 2299–2304. 10.1021/jacs.8b08687. 30648388

[ref19] Tavani F.; Martini A.; Capocasa G.; Di Stefano S.; Lanzalunga O.; D’Angelo P. Direct Mechanistic Evidence for a Non-Heme Complex Reaction through a Multivariate XAS Analysis. Inorg. Chem. 2020, 59, 9979–9989. 10.1021/acs.inorgchem.0c01132. 32598841 PMC8008396

[ref20] Busato M.; Melchior A.; Migliorati V.; Colella A.; Persson I.; Mancini G.; Veclani D.; D’Angelo P. Elusive Coordination of the Ag^+^ Ion in Aqueous Solution: Evidence for a Linear Structure. Inorg. Chem. 2020, 59, 17291–17302. 10.1021/acs.inorgchem.0c02494. 33233885

[ref21] Isegawa K.; Ueda K.; Hiwasa S.; Amemiya K.; Mase K.; Kondoh H. Formation of Carbonate on Ag(111) under Exposure to Ethylene and Oxygen Gases Evidenced by Near Ambient Pressure XPS and NEXAFS. Chem. Lett. 2019, 48, 159–162. 10.1246/cl.180891.

[ref22] Castán-Guerrero C.; Krizmancic D.; Bonanni V.; Edla R.; Deluisa A.; Salvador F.; Rossi G.; Panaccione G.; Torelli P. A Reaction Cell for Ambient Pressure Soft X-ray Absorption Spectroscopy. Rev. Sci. Instrum. 2018, 89, 054101 10.1063/1.5019333. 29864817

[ref23] Braglia L.; Fracchia M.; Ghigna P.; Minguzzi A.; Meroni D.; Edla R.; Vandichel M.; Ahlberg E.; Cerrato G.; Torelli P. Understanding Solid-Gas Reaction Mechanisms by Operando Soft X-Ray Absorption Spectroscopy at Ambient Pressure. J. Phys. Chem. C 2020, 124, 14202–14212. 10.1021/acs.jpcc.0c02546. PMC8008446 33815647

[ref24] Braglia L.; Tavani F.; Mauri S.; Edla R.; Krizmancic D.; Tofoni A.; Colombo V.; D’Angelo P.; Torelli P. Catching the Reversible Formation and Reactivity of Surface Defective Sites in Metal–Organic Frameworks: An Operando Ambient Pressure-NEXAFS Investigation. J. Phys. Chem. Lett. 2021, 12, 9182–9187. 10.1021/acs.jpclett.1c02585. 34528795 PMC9282676

[ref25] Tavani F.; Fracchia M.; Tofoni A.; Braglia L.; Jouve A.; Morandi S.; Manzoli M.; Torelli P.; Ghigna P.; D’Angelo P. Structural and Mechanistic Insights into Low-Temperature CO Oxidation over a Prototypical High Entropy Oxide by Cu L-edge Operando Soft X-ray absorption Spectroscopy. Phys. Chem. Chem. Phys. 2021, 23, 26575–26584. 10.1039/D1CP03946F. 34812450

[ref26] Martini A.; Guda S.; Guda A.; Smolentsev G.; Algasov A.; Usoltsev O.; Soldatov M.; Bugaev A.; Rusalev Y.; Lamberti C.; Soldatov A. PyFitit: The Software for Quantitative Analysis of XANES Spectra Using Machine-Learning Algorithms. Comput. Phys. Commun. 2019, 107064.

[ref27] Martini A.; Guda A. A.; Guda S. A.; Dulina A.; Tavani F.; D’Angelo P.; Borfecchia E.; Soldatov A. V. In Synchrotron Radiation Science and Applications. Springer Proceedings in Physics; Di Cicco A., Giuli G., Trapananti A., Eds.; Springer, 2021; Vol. 220; pp 65–84.

[ref28] Tavani F.; Capocasa G.; Martini A.; Sessa F.; Di Stefano S.; Lanzalunga O.; D’Angelo P. Direct Structural and Mechanistic Insights into Fast Bimolecular Chemical Reactions in Solution through a Coupled XAS/UV-Vis Multivariate Statistical Analysis. Dalton Trans 2021, 50, 131–142. 10.1039/D0DT03083J. 33284934

[ref29] Tavani F.; Capocasa G.; Martini A.; Sessa F.; Di Stefano S.; Lanzalunga O.; D’Angelo P. Activation of C-H Bonds by a Nonheme Iron(IV)-Oxo Complex: Mechanistic Evidence through a Coupled EDXAS/UV-Vis Multivariate Analysis. Phys. Chem. Chem. Phys. 2021, 23, 1188–1196. 10.1039/D0CP04304D. 33355324

[ref30] Tavani F.; Fracchia M.; Pianta N.; Ghigna P.; Quartarone E.; D’Angelo P. Multivariate Curve Resolution Analysis of Operando XAS Data for the Investigation of the Lithiation Mechanisms in High Entropy Oxides. Chem. Phys. Lett. 2020, 760, 137968 10.1016/j.cplett.2020.137968.

[ref31] Kleiman S.; Chaim R. Thermal Stability of MgO Nanoparticles. Mater. Lett. 2007, 61, 4489–4491. 10.1016/j.matlet.2007.02.032.

[ref32] Aritani H.; Yamada H.; Nishio T.; Shiono T.; Imamura S.; Kudo M.; Hasegawa S.; Tanaka T.; Yoshida S. Characterization of Li-Doped MgO Catalysts for Oxidative Coupling of Methane by Means of Mg K-Edge XANES. J. Phys. Chem. B 2000, 104, 10133–10143. 10.1021/jp000291y.

[ref33] Klysubun W.; Kidkhunthod P.; Tarawarakarn P.; Sombunchoo P.; Kongmark C.; Limpijumnong S.; Rujirawat S.; Yimnirun R.; Tumcharern G.; Faungnawakij K. SUT-NANOTEC-SLRI Beamline for X-ray Absorption Spectroscopy. J. Synchr. Rad. 2017, 24, 707–716. 10.1107/S1600577517004830. 28452765

[ref34] Singh J.; Kumar M.; Lee I.-J.; Chae K. X-ray Reflectivity and Near Edge X-ray Absorption Fine Structure Investigations of MgO Thin Films. Appl. Sci. Lett. 2017, 3.

[ref35] Yoshioka S.; Tsuruta K.; Yamamoto T.; Yasuda K.; Matsumura S.; Ishikawa N.; Kobayashi E. X-ray Absorption Near Edge Structure and First-Principles Spectral Investigations of Cationic Disorder in MgAl_2_O_4_ Induced by Swift Heavy Ions. Phys. Chem. Chem. Phys. 2018, 20, 4962–4969. 10.1039/C7CP07591J. 29387834

[ref36] Witte K.; Streeck C.; Mantouvalou I.; Suchkova S. A.; Lokstein H.; Grötzsch D.; Martyanov W.; Weser J.; Kanngieer B.; Beckhoff B.; Stiel H. Magnesium K-Edge NEXAFS Spectroscopy of Chlorophyll A in Solution. J. Phys. Chem. B 2016, 120, 11619–11627. 10.1021/acs.jpcb.6b05791. 27783515

[ref37] Joly Y. X-ray Absorption Near-Edge Structure Calculations Beyond the Muffin-Tin Approximation. Phys. Rev. B 2001, 63, 125120 10.1103/PhysRevB.63.125120.

[ref38] Bunău O.; Joly Y. Self-Consistent Aspects of X-ray Absorption Calculations. J. Phys: Condens. Matter 2009, 21, 345501. 21715786 10.1088/0953-8984/21/34/345501

[ref39] Guda S. A.; Guda A. A.; Soldatov M. A.; Lomachenko K. A.; Bugaev A. L.; Lamberti C.; Gawelda W.; Bressler C.; Smolentsev G.; Soldatov A. V.; Joly Y. Optimized Finite Difference Method for the Full-Potential XANES Simulations: Application to Molecular Adsorption Geometries in MOFs and Metal-Ligand Intersystem Crossing Transients. J. Chem. Th. Comp. 2015, 11, 4512–4521. 10.1021/acs.jctc.5b00327. 26575941

[ref40] Pankin I.; Borfecchia E.; Martini A.; Lomachenko K.; Lamberti C.; Soldatov A. DFT-assisted XANES Simulations to Discriminate Different Monomeric Cu^*II*^ Species in CHA Catalysts. Radiat. Phys. Chem. 2020, 175, 108510 10.1016/j.radphyschem.2019.108510.

[ref41] Amodeo J.; Merkel S.; Tromas C.; Carrez P.; Korte-Kerzel S.; Cordier P.; Chevalier J. Dislocations and Plastic Deformation in MgO Crystals: A Review. Crystals 2018, 8, 240 10.3390/cryst8060240

[ref42] Becke A. D. A New Mixing of Hartree-Fock and Local Density Functional Theories. J. Chem. Phys. 1993, 98, 1372–1377. 10.1063/1.464304.

[ref43] Lee C.; Yang W.; Parr R. G. Development of the Colle-Salvetti Correlation-Energy Formula into a Functional of the Electron Density. Phys. Rev. B 1988, 37, 785–789. 10.1103/PhysRevB.37.785. 9944570

[ref44] Frisch M. J.; Trucks G. W.; Schlegel H. B.; Scuseria G. E.; Robb M. A.; Cheeseman J. R.; Scalmani G.; Barone V.; Mennucci B.; Petersson G. A.; Nakatsuji H.; Caricato M.; Li X.; Hratchian H. P.; Izmaylov A. F.; Bloino J.; Zheng G.; Sonnenberg J. L.; Hada M.; Ehara M.; Toyota K.; Fukuda R.; Hasegawa J.; Ishida M.; Nakajima T.; Honda Y.; Kitao O.; Nakai H.; Vreven T.; Montgomery J. A. Jr.; Peralta J. E.; Ogliaro F.; Bearpark M.; Heyd J. J.; Brothers E.; Kudin K. N.; Staroverov V. N.; Kobayashi R.; Normand J.; Raghavachari K.; Rendell A.; Burant J. C.; Iyengar S. S.; Tomasi J.; Cossi M.; Rega N.; Millam J. M.; Klene M.; Knox J. E.; Cross J. B.; Bakken V.; Adamo C.; Jaramillo J.; Gomperts R.; Stratmann R. E.; Yazyev O.; Austin A. J.; Cammi R.; Pomelli C.; Ochterski J. W.; Martin R. L.; Morokuma K.; Zakrzewski V. G.; Voth G. A.; Salvador P.; Dannenberg J. J.; Dapprich S.; Daniels A. D.; Farkas Ã.; Foresman J. B.; Ortiz J. V.; Cioslowski J.; Fox D. J. Gaussian 09, Revision A.02, 2009.

[ref45] Pye C. C.; Rudolph W. W. An Ab Initio and Raman Investigation of Magnesium(II) Hydration. J. Phys. Chem. A 1998, 102, 9933–9943. 10.1021/jp982709m.

[ref46] Matwiyoff N. A.; Taube H. Direct Determination of the Solvation Number of Magnesium(II) ion in water, Aqueous Acetone, and Methanolic Acetone solutions. J. Am. Chem. Soc. 1968, 90, 2796–2800. 10.1021/ja01013a012.

[ref47] Skipper N. T.; Neilson G. W.; Cummings S. C. An X-ray Diffraction Study of Ni(aq)^2+^ and Mg(aq)^2+^ by Difference Methods. J. Phys.: Condensed Matter 1989, 1, 3489–3506.

[ref48] Bernal-Uruchurtu M. I.; Ortega-Blake I. A Refined Monte Carlo Study of Mg^2+^ and Ca^2+^ Hydration. J. Chem. Phys. 1995, 103, 1588–1598. 10.1063/1.469781.

[ref49] Obst S.; Bradaczek H. Molecular Dynamics Study of the Structure and Dynamics of the Hydration Shell of Alkaline and Alkaline-Earth Metal Cations. J. Phys. Chem. 1996, 100, 15677–15687. 10.1021/jp961384b.

[ref50] Tongraar A.; Michael Rode B. The Role of Non-Additive Contributions on the Hydration Shell Structure of Mg^2+^ Studied by Born-Oppenheimer Ab Initio Quantum Mechanical/Molecular Mechanical Molecular Dynamics Simulation. Chem. Phys. Lett. 2001, 346, 485–491. 10.1016/S0009-2614(01)00923-X.

[ref51] Tongraar A.; Rode B. M. Structural Arrangement and Dynamics of the Hydrated Mg^2+^: an Ab Initio QM/MM Molecular Dynamics Simulation. Chem. Phys. Lett. 2005, 409, 304–309. 10.1016/j.cplett.2005.04.062.

[ref52] Dudev T.; Lim C. Incremental Binding Free Energies in Mg^2+^ Complexes: A DFT Study. J. Phys. Chem. A 1999, 103, 8093–8100. 10.1021/jp991575p.

[ref53] D’Angelo P.; Migliorati V.; Sessa F.; Mancini G.; Persson I. XANES Reveals the Flexible Nature of Hydrated Strontium in Aqueous Solution. J. Phys. Chem. B 2016, 120, 4114–4124. 10.1021/acs.jpcb.6b01054. 27065305

[ref54] Spezia R.; Duvail M.; Vitorge P.; Cartailler T.; Tortajada J.; D’Angelo P.; Gaigeot M.-P.; Chillemi G. A Coupled Car-Parrinello Molecular Dynamics and EXAFS Data Analysis Investigation of Aqueous Co^2+^. J. Phys. Chem. A 2006, 110, 13081–13088. 10.1021/jp064688z. 17134169

[ref55] D’Angelo P.; Roscioni O. M.; Chillemi G.; Della Longa S.; Benfatto M. Detection of Second Hydration Shells in Ionic Solutions by XANES: Computed Spectra for Ni^2+^ in Water Based on Molecular Dynamics. J. Am. Chem. Soc. 2006, 128, 1853–1858. 10.1021/ja0562503. 16464084

[ref56] Radnai T.; Kálmán E.; Pollmer K. X-Ray Diffraction Study of MgCl_2_ in Methanol. Zeitschrift fu̅r Naturforschung A 1984, 39, 464–470. 10.1515/zna-1984-0508.

[ref57] Nakamura S.; Meiboom S. Proton Magnetic Resonance Studies of the Solvation Shell of Mg^2+^ in Methanol. Solvation Number and Exchange Rate. J. Am. Chem. Soc. 1967, 89, 1765–1772. 10.1021/ja00984a001.

[ref58] Faralli C.; Pagliai M.; Cardini G.; Schettino V. Ab Initio Molecular Dynamics Study of Mg^2+^ and Ca^2+^ Ions in Liquid Methanol. J. Chem. Th. Comp. 2008, 4, 156–163. 10.1021/ct700209v. 26619989

