# Supplementary material for: Caught while Dissolving: Revealing the Interfacial Solvation of the Mg2+ Ions on the MgO Surface
Source: ACS Appl Mater Interfaces. 2022 Aug 15;14(33):38370–8. doi: 10.1021/acsami.2c10005 (PMC9412945; doi:10.1021/acsami.2c10005)
Supplement: Supplementary file 1 — am2c10005_si_001.pdf [file am2c10005_si_001.pdf]

Supporting Information

Caught while dissolving: revealing the interfacial solvation of the Mg^{2+} ions on the MgO surface

Francesco Tavani[*,†], Matteo Busato[†], Luca Braglia[‡], Silvia Mauri[‡,§], Piero Torelli[‡], Paola D'Angelo[*,†]

[†] Dipartimento di Chimica, Università di Roma "La Sapienza", P.le A. Moro 5, 00185 Roma, Italy.
[‡] CNR - Istituto Officina dei Materiali, TASC, I-34149 Trieste, Italy.
[§] Dipartimento di Fisica, Università di Trieste, Via A. Valerio 2, 34127 Trieste, Italy.
[*] p.dangelo@uniroma1.it, francesco.tavani@uniroma1.it

1 Ambient Pressure Near Edge X-ray Absorption Fine Structure measurements

Table S1: Temperature and time recording of each of the AP-NEXAFS Mg K-edge spectra (shown in Fig. 1a of the main text) recorded during the experiment involving the exposure of MgO to water.

Scan number	Temperature (°C)	Time (min)
1	50(2)	0
2	50(2)	5
3	50(2)	10
4	50(2)	15
5	50(2)	20
6	50(2)	25
7	50(2)	30
8	50(2)	35
9	50(2)	40
10	50(2)	45
11	50(2)	50
12	50(2)	55
13	50(2)	60
14	50(2)	65
15	50(2)	70
16	50(2)	75
17	50(2)	80
18	50(2)	85
19	50(2)	90
20	50(2)	95
21	100(2)	110
22	150(2)	125
23	200(2)	140
24	250(2)	155

Table S2: Temperature and time recording of each of the AP-NEXAFS Mg K-edge spectra (shown in Fig. 1b of the main text) recorded during the experiment involving the exposure of MgO to methanol.

Scan number	Temperature (°C)	Time (min)
1	50(2)	0
2	50(2)	5
3	50(2)	10
4	50(2)	15
5	50(2)	20
6	50(2)	25
7	50(2)	30
8	50(2)	35
9	50(2)	40
10	50(2)	45
11	50(2)	50
12	50(2)	55
13	50(2)	60
14	50(2)	65
15	50(2)	70
16	50(2)	75
17	50(2)	80
18	83(2)	85
19	116(2)	90
20	149(2)	95
21	182(2)	100
22	215(2)	105
23	250(2)	110

2 Processing of AP-NEXAFS spectra

All measured Mg K-edge AP-NEXAFS spectra were subjected to background subtraction using the SNIP algorithm for background estimation,[1] and subsequently normalized dividing each background subtracted spectrum by its maximum intensity. Examples of raw experimental spectra, SNIP backgrounds and background corrected spectra are shown in Figure S2.

3 Decomposition of the AP-NEXAFS data into the spectra and fractional concentrations of key components

Time-resolved spectroscopical measurements of chemical processes yield a series of spectra that may be positioned in a matrix $\boldsymbol{D}$, where the columns of $\boldsymbol{D}$ are the spectra measured at time t. According to Lambert-Beer's law, at any given time a number N of "pure" and independent components weighed by their fractional concentration contributes to the measured signal.[2] Decomposing the experimental data into the spectra associated to the key species and in their relative concentration profiles can offer important insight in the investigated process. In the present work, such decomposition was performed with the PyFitit code,[2] a software that uses to such end an algorithm belonging to the MCR family.
The starting point is the Singular Value Decomposition (SVD) equation:

$$\boldsymbol{D} = \boldsymbol{U} \cdot \boldsymbol{\Sigma} \cdot \boldsymbol{V} + \boldsymbol{E} \tag{1}$$

where the product $\boldsymbol{U} \cdot \boldsymbol{\Sigma}$ contains, on its N columns, a set of values that may be associated to the normalized absorption coefficients, $\boldsymbol{\Sigma}$ is a diagonal matrix known as the *singular values* term, whose elements are sorted in decreasing order, while $\boldsymbol{V}$ can be interpreted as the concentration matrix associated to the N-selected components. Lastly, the error matrix $\boldsymbol{E}$ represents the lack of fit between the experimental data matrix $\boldsymbol{D}$ and the reconstructed matrix $\boldsymbol{\mu} = \boldsymbol{U} \cdot \boldsymbol{\Sigma} \cdot \boldsymbol{V}$. The SVD deconvolution depends on the correct estimation of the number of components N present in the experimental spectral matrix. To this end, in this investigation we evaluated the percentage error committed in reproducing the experimental data with an increasing number N of components, as detailed in the main text, as shown in Figure S4. The percentage error function has been calculated with the following expression:

$$R(n) = \frac{\sum_{i=1}^{K} \sum_{j=1}^{m} |d_{ij} - \mu_{ij}^{PC=n}|}{\sum_{i=1}^{K} \sum_{j=1}^{m} |d_{ij}|} \times 100 \tag{2}$$

where d_{ij} and $\mu_{ij}^{PC=n}$ are the normalized absorbance values for the dataset and for the dataset reconstructed with N = n, respectively (K and m represent the number of acquired spectra and of the energy points, respectively, while $n = 1, 2, ...K$).
At this point, all matrices in Equation 1 are solely mathematical solutions to the decomposition problem without physico-chemical meaning. Once N is established, the approach implemented by PyFitIt requires the introduction of a transformation N × N matrix $\boldsymbol{T}$ in Equation 1, using the relation $\boldsymbol{I} = \boldsymbol{T} \cdot \boldsymbol{T}^{-1}$:

$$\boldsymbol{D} = \boldsymbol{U} \cdot \boldsymbol{\Sigma} \cdot \boldsymbol{T} \cdot \boldsymbol{T}^{-1} \cdot \boldsymbol{V} + \boldsymbol{E} \tag{3}$$

where the spectra belonging to the key species are given by $\boldsymbol{S} = \boldsymbol{U} \cdot \boldsymbol{\Sigma} \cdot \boldsymbol{T}$ and their concentration profiles by $\boldsymbol{C} = \boldsymbol{T}^{-1} \cdot \boldsymbol{V}$. Subsequently, the matrix elements T_{ij} of matrix $\boldsymbol{T}$ are modified by

sliders to achieve $\boldsymbol{S}$ and $\boldsymbol{C}$ which are chemically and physically interpretable. Once this step is achieved, one can finally write:

$$\boldsymbol{D} = \boldsymbol{S} \cdot \boldsymbol{C} + \boldsymbol{E} \tag{4}$$

The unknown number of T_{ij} elements of $\boldsymbol{T}$ is in principle equal to N^2. In order to reduce such ambiguity, the AP-NEXAFS measured on the clean MgO surface was constrained to coincide with the first extracted spectral component for the analysis of the data related to the exposure of the MgO surface both to H_2O and MeOH. This operation allows the reduction of the number of unknown T_{ij} elements from N^2 to $N^2 - N$.

In the case of the H_2O process, a 2 × 2 matrix $\boldsymbol{T}_{water}$ was defined containing four elements. The solution for the decomposition presented in Equation 4 was obtained using the following matrix:

$$\boldsymbol{T}_{water} = \begin{pmatrix} T_{11} & -0.168 \\ T_{21} & -3.198 \end{pmatrix} \tag{5}$$

Conversely, the following 2 × 2 matrix $\boldsymbol{T}_{methanol}$ was defined, containing four elements, to decompose the UV–Vis data relative to the MeOH process:

$$\boldsymbol{T}_{methanol} = \begin{pmatrix} T_{11} & -0.0110 \\ T_{21} & -0.0468 \end{pmatrix} \tag{6}$$

4 Molecular dynamics simulations

Classical MD simulations were carried out on the Mg^{2+} ion in aqueous solution and in methanol. Cubic boxes with one Mg^{2+} ion and 500 water or MeOH molecules were built with side lengths of respectively 24.68 and 32.28 Å, chosen in order to reproduce pure water and MeOH densities. Structures and interactions of the chemical species were represented with the SPC/E model for water,[3] while the OPLS force field was employed for MeOH.[4] Van der Waals interactions were introduced with the Lennard-Jones (LJ) potential with cross terms calculated with the Lorentz-Berthelot combining rules. The Mg^{2+} ion was represented with LJ parameters optimized by Babu and Lim for the water case ($\sigma = 2.430$ Å, $\epsilon = 0.0266$ kcal mol^{-1}).[5] A cut-off radius of 12 Å was employed for all the non-bonded interactions and long-range electrostatic forces were taken into account with the Particle Mesh Ewald method.[6,7]

Initial configurations were build with random positions with the PACKMOL package.[8] After energy minimization, each system was simulated in NVT conditions at 323 K for 10 ns, with the first 2 ns discarded as equilibration time. The temperature was controlled with the Nosé-Hoover thermostat with a coupling constant of 0.5 ps. Equations of motion were integrated with the leap-frog algorithm using a 1 fs time-step, while trajectories were saved every 100 steps. Stretching vibrations involving hydrogen atoms were constrained with the LINCS algorithm.[9] Simulations were performed with the Gromacs 2020.6 program.[10]

5 DFT calculations

Minimum energy structures of clusters formed by the Mg^{2+} ion with 4 and 6 water/MeOH molecules have been optimized at the density functional theory (DFT) level in gas phase. The B3LYP functional[11,12] was employed with the gaussian-type 6-31G(d,p) basis set for all the elements. Vibrational analysis was carried out to confirm the absence of imaginary frequencies and

check that the stationary points were true minima. Calculations were carried out with the Gaussian 09 program.[13]

Table S3: Comparison of the average Mg–O distances obtained for the $[Mg(H_2O)_n]^{2+}$ and $[Mg(MeOH)_n]^{2+}$ ($n = 4, 6$) clusters optimized at the DFT level with the bond lengths reported in the literature.

Species	n	$d_{(Mg-O)}$ (Å) This work	$d_{(Mg-O)}$ (Å) Literature[14]
1. $[Mg(H_2O)_4]^{2+}$	4	2.01	
2. $[Mg(H_2O)_6]^{2+}$	6	2.10	2.110
3. $[Mg(MeOH)_4]^{2+}$	4	2.00	
4. $[Mg(MeOH)_6]^{2+}$	6	2.12	2.118

6 Theoretical NEXAFS simulations

The Mg K-edge absoption spectra presented in this study were calculated using the FDMNES code, implementing the recently developed sparse solver method.[15–17] This software is based upon the Finite Difference Method (FDM), an attractive approach for the simulation of the photoelectron wave function beyond 100 eV above the absoption edge, avoiding the muffin tin approximation used in many common multiple scattering theory based codes. Specifically, the unit cell-normalized cross section $\sigma(\omega)$ was calculated as:

$$\sigma(\omega) = 4\pi^2\alpha\hbar\omega \sum_j \sum_{f,g} |\Psi_f|\Theta|\Psi_g^{(j)}|^2\delta(\hbar\omega - (E - E_g^{(j)})) \tag{7}$$

where $\hbar\omega$ is the energy of the photon, α the fine structure constant, E_g and E are the energies of the ground state $\Psi_g^{(j)}$ and Ψ_f, respectively, while the summation over j includes the contribution of all the atoms in the unit cells possessing index j.[18] The electron - photon interaction is treated classically employing the operator Θ, neglecting the magnetic part of the electromagnetic field and describing its electring portion with the first two terms of the multipolar expansion (corresponding to electric dipole and electric quadrupole excitations):

$$\Theta = \boldsymbol{\epsilon} \cdot \boldsymbol{r}(1 + \frac{i}{2}\boldsymbol{k} \cdot \boldsymbol{r}) \tag{8}$$

where $\boldsymbol{r}$ is the relative position from the photoabsorber, $\boldsymbol{\epsilon}$ is the photon polarization and $\boldsymbol{k}$ the photon wave vector. In all calculations the Schrōdinger-like equation was solved self-consistently to find the final states where there is a transition.[18]

The calculated cross-sections were convoluted in a post-processing step by an energy-dependent arctangent function (Γ) in order to compare them to the experimental NEXAFS data. Γ is defined as follows:

$$\Gamma = \Gamma_i + \Gamma_f(\frac{1}{2} + \frac{1}{\pi}arctan(\frac{\pi\Gamma_f}{3E_w}(\frac{\boldsymbol{E} - E_f}{E_c} - \frac{E_c^2}{(\boldsymbol{E} - E_f)^2}))) \tag{9}$$

where $\boldsymbol{E}$ is the energy scale of the Mg K–edge NEXAFS spectrum, Γ_i and Γ_f are the core-level and final-state widths, respectively, E_c and E_w are the center and width of the arctangent function, respectively, while E_f is the Fermi energy.[19] The density of electronic states of the investigated systems was also calculated through FDMNES. In order to reproduce the local environment of the Mg^{2+} ion at the surface of MgO within a sufficient number of atomic planes, as probed by the AP-NEXAFS technique, the NEXAFS spectrum of MgO was simulated including in the calculation all scattering atoms within a cutoff radius of 5Å. The NEXAFS spectra obtained from the MD-extracted configurations were simulated including in the calculations water and methanol molecules within 6 Å of the photoabsorber, i.e. until convergence was reached.
A positive shift of 1294.9 eV was applied to all theoretical NEXAFS simulated spectra for the alignement with the experimental data.

7 Supplementary Figures S1–S9

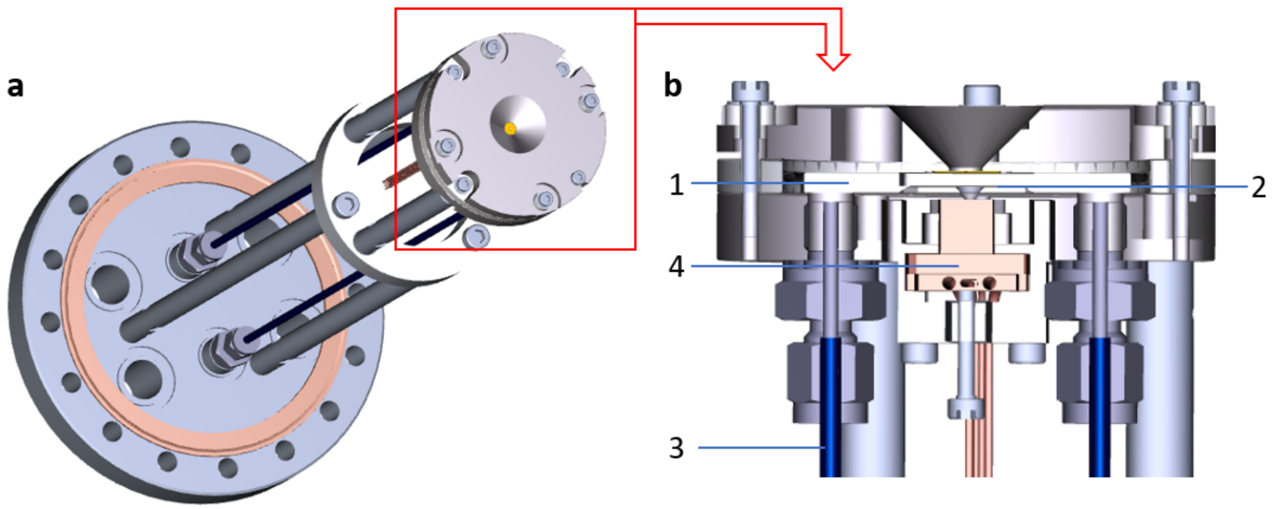

Figure S1: a) 3D rendering of the reaction cell developed at APE-HE for the *operando* NEXAFS experiment. b) Side view of the reaction cell.

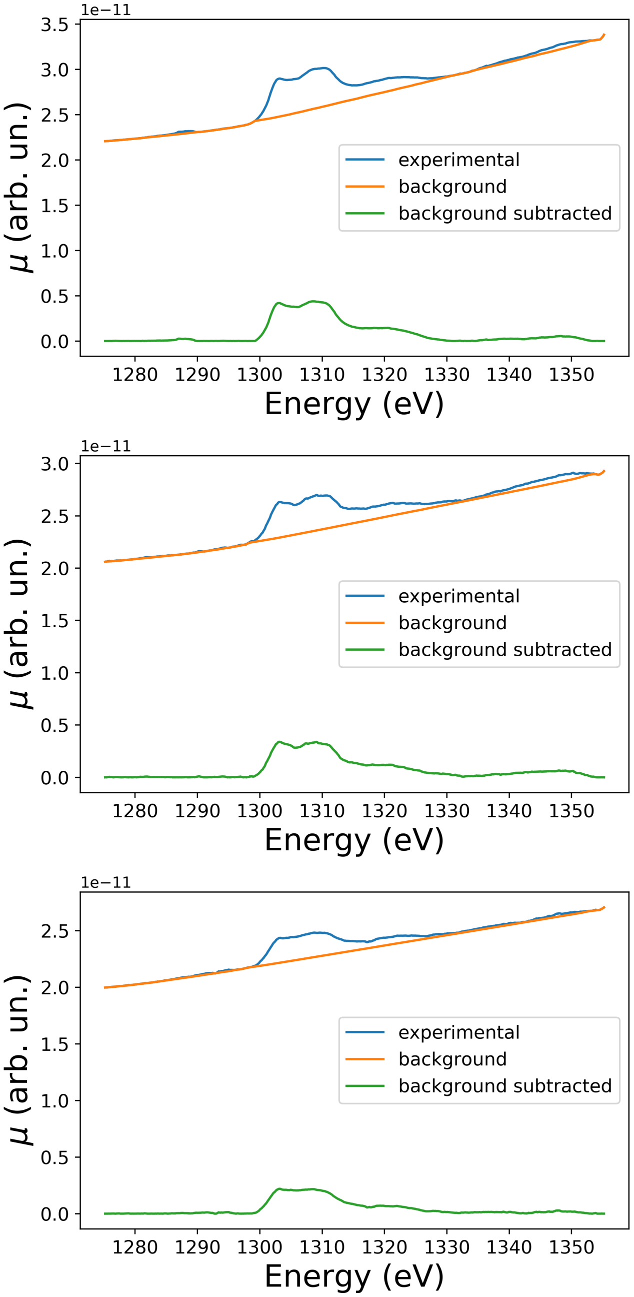

Figure S2: Examples of the background subtraction procedure applied in this study to the raw measured Mg K-edge AP-NEXAFS spectra. The raw spectra, SNIP estimated backgrounds and background subtracted spectra are portrayed with blue, orange and green full lines, respectively.

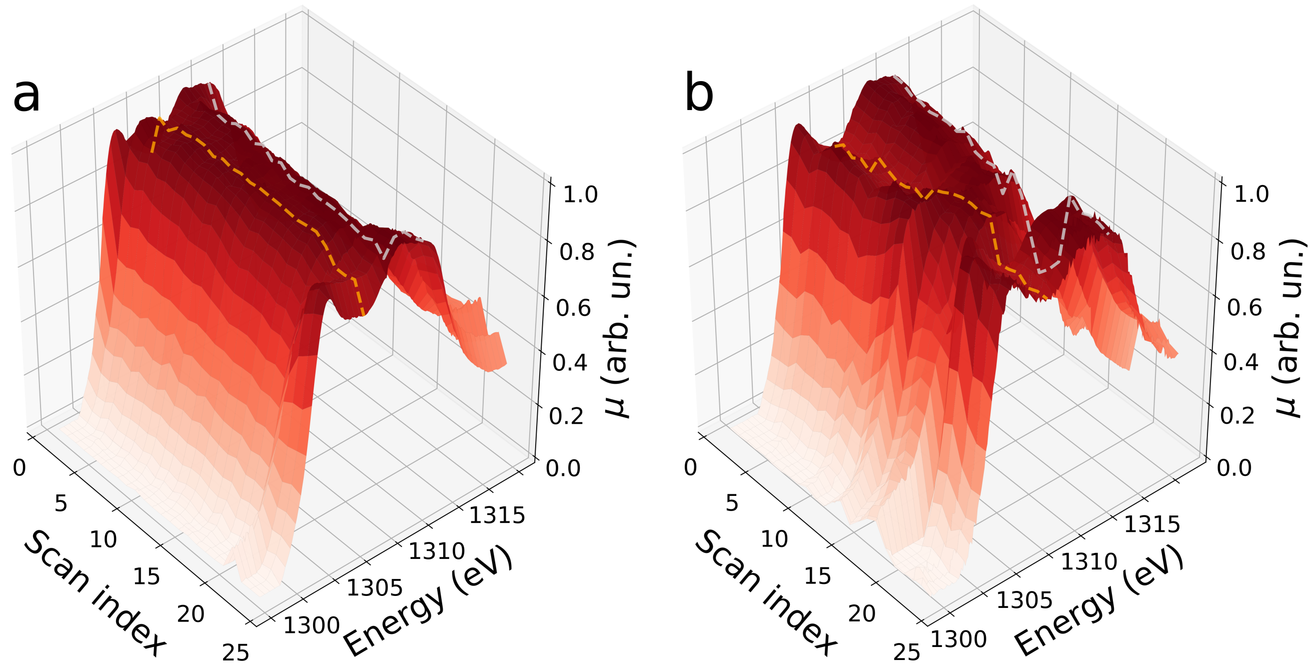

Figure S3: Evolution of the Mg K–edge AP-NEXAFS spectra of MgO upon exposure to water (a) and methanol (b) at 50°C. In both panels constant energy cuts are drawn at 1305.7 eV (yellow dotted lines) and at 1310.2 eV (grey dotted lines).

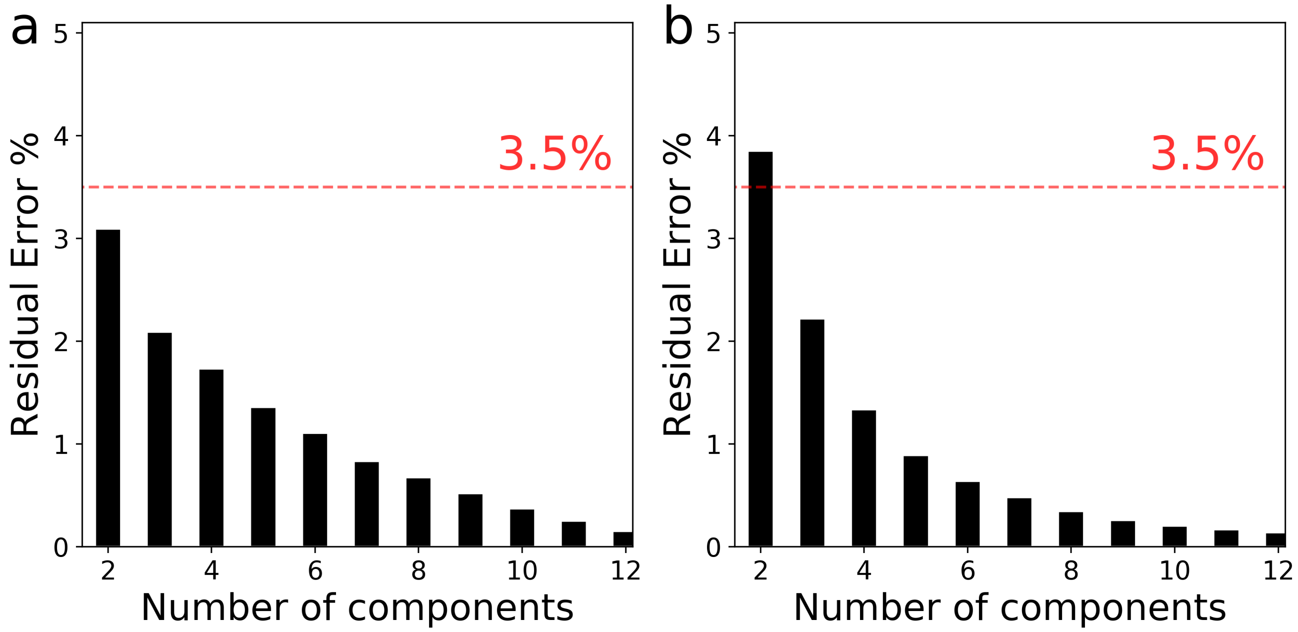

Figure S4: Percentage error committed in reconstructing the AP-NEXAFS spectra measured upon exposure of the MgO surface to H_2O (a) and MeOH (b) at 50°C, as a function of increasing number of components. The average percentage error value of 3.5% committed in reproducing the data using 2 components is evidenced in both panels to aide the reader in the visualization.

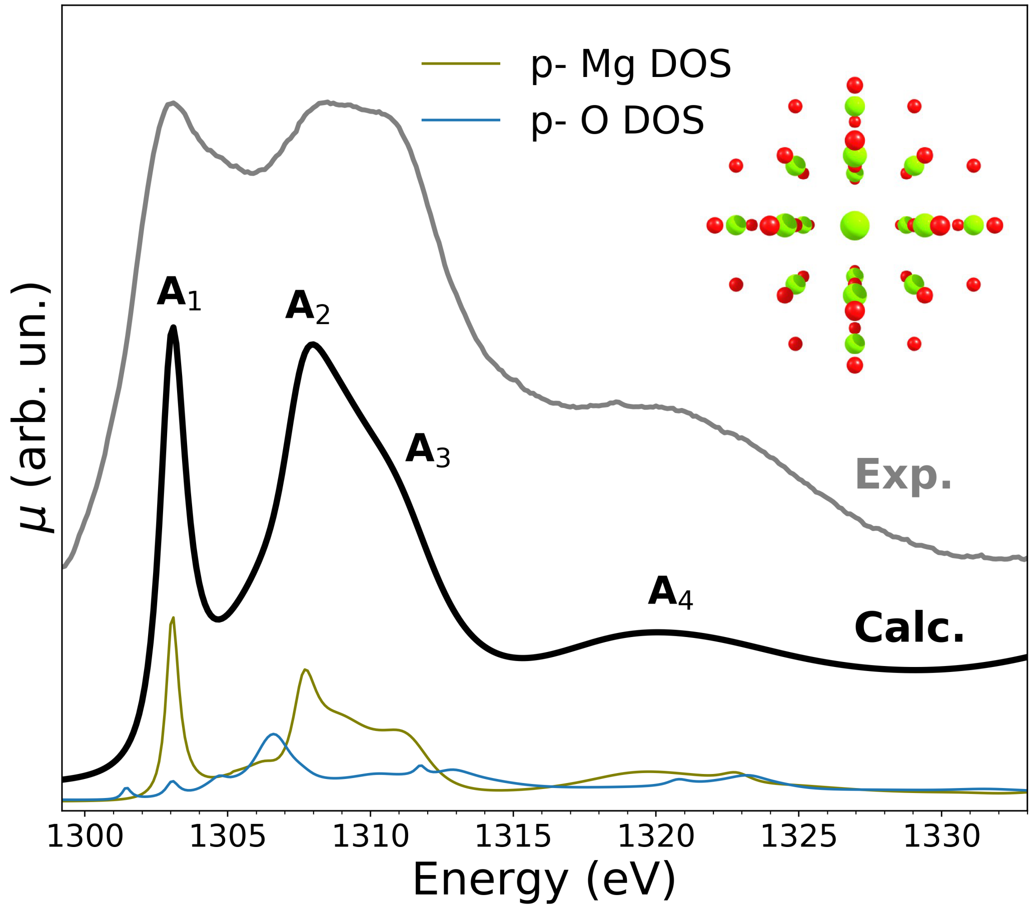

Figure S5: Mg K-edge NEXAFS simulated spectrum of MgO (full black line), together with the calculated Mg- and O- partial density of *p* states and the associated molecular cluster. The experimental MCR-extracted curve (full grey line) is presented above the theoretical one.

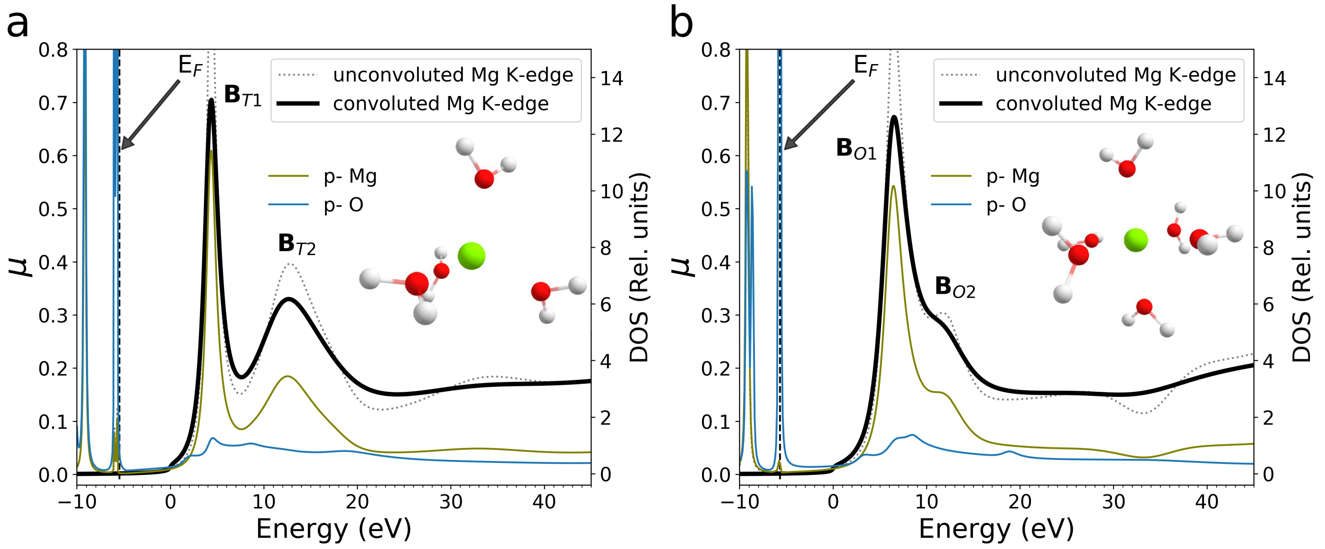

Figure S6: Mg K-edge NEXAFS simulated spectra of DFT-optimized tetrahedral $[Mg(H_2O)_4]^{2+}$ (a) and octahedral $[Mg(H_2O)_6]^{2+}$ (b) complexes. The theoretical spectra before (dashed black lines) and after (full black lines) convolution are presented, together with the calculated Mg- and O- partial density of p states and the associated molecular clusters. The calculated Fermi energy levels of the $[Mg(H_2O)_4]^{2+}$ and $[Mg(H_2O)_6]^{2+}$ species are equal to -5.45 eV and to -5.68 eV, respectively, and represent the borders of the dotted vertical black lines in the two panels.

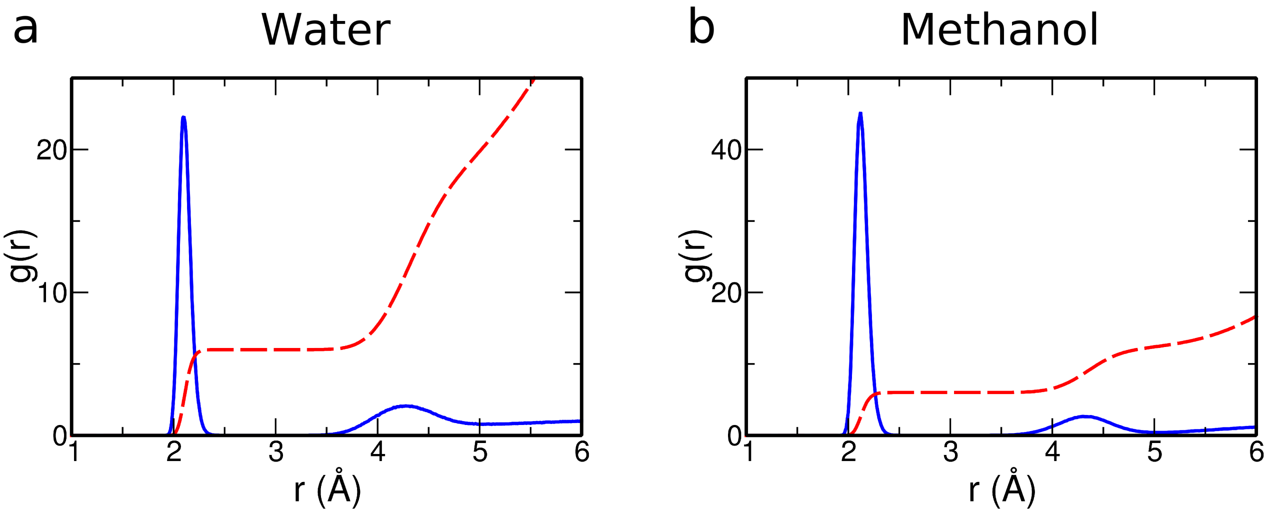

Figure S7: Mg-O radial distribution functions *g(r)'s* (blue solid lines) and corresponding running integration numbers (red dashed lines) calculated from MD simulations of Mg^{2+} in A) water and B) methanol.

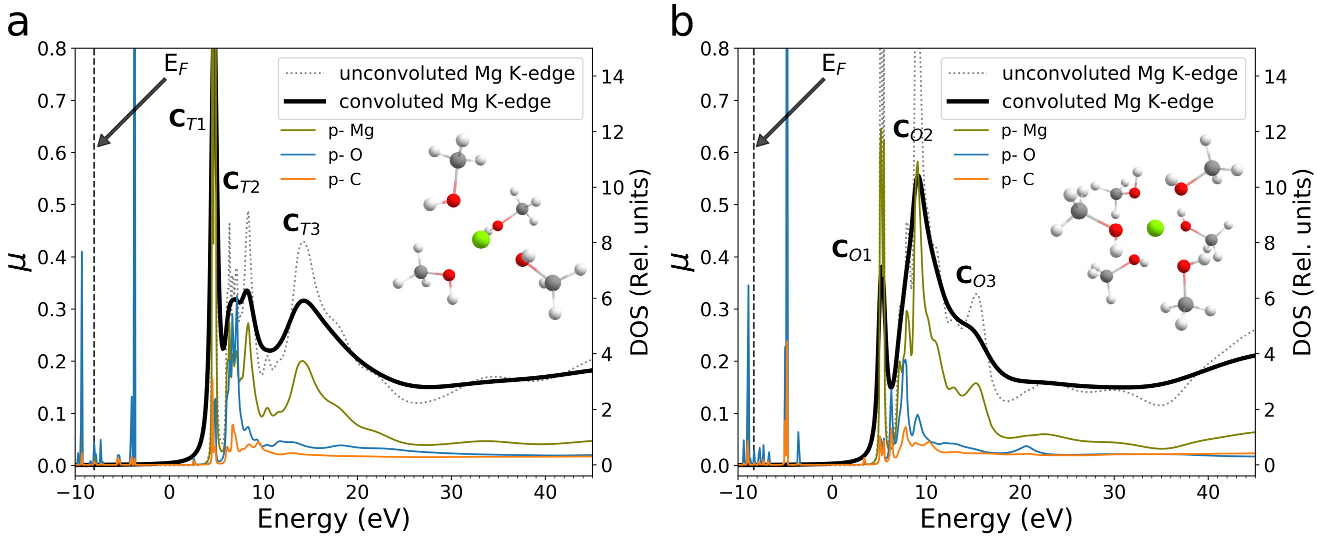

Figure S8: Mg K-edge NEXAFS simulated spectra of DFT-optimized tetrahedral $[Mg(MeOH)_4]^{2+}$ (a) and octahedral $[Mg(MeOH)_6]^{2+}$ (b) complexes. The theoretical spectra before (dashed black lines) and after (full black lines) convolution are presented, together with the calculated Mg-, O- and C- partial density of p states and the associated molecular clusters. The calculated Fermi energy levels of the $[Mg(MeOH)_4]^{2+}$ and $[Mg(MeOH)_6]^{2+}$ species are equal to -7.98 eV and to -8.34 eV, respectively, and represent the borders of the dotted vertical black lines in the two panels.

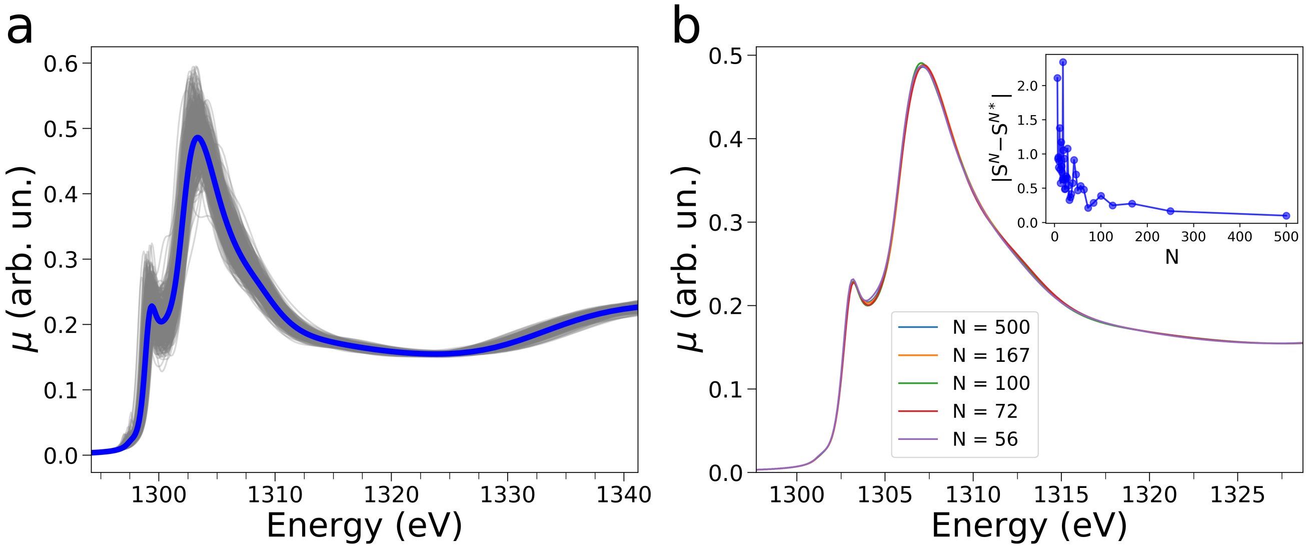

Figure S9: Theoretical NEXAFS spectra (grey full lines) calculated for MD snapshots of the Mg^{2+} ion in methanol at 50°C and converged NEXAFS average (blue full line) of 500 spectra (a) along with a selection of average NEXAFS spectra calculated with a variable number (N) of spectra (b). The associated evolutions of the total absolute differences between averages of spectra computed with increasing N values are shown in the insets of panel.

References

[1] Tomoyori, K.; Hirano, Y.; Kurihara, K.; Tamada, T. *Journal of Physics: Conference Series* **2015**, *664*, 072049.

[2] Martini, A.; Guda, S.; Guda, A.; Smolentsev, G.; Algasov, A.; Usoltsev, O.; Soldatov, M.; Bugaev, A.; Rusalev, Y.; Lamberti, C.; Soldatov, A. *Comput. Phys. Comm.* **2019**, 107064.

[3] Berendsen, H. J. C.; Grigera, J. R.; Straatsma, T. P. *J Phys Chem* **1987**, *91*, 6269–6271.

[4] Jorgensen, W. L.; Maxwell, D. S.; Tirado-Rives, J. *J Am Chem Soc* **1996**, *118*, 11225–11236.

[5] Babu, C. S.; Lim, C. *J Phys Chem A* **2006**, *110*, 691–699.

[6] Darden, T.; York, D.; Pedersen, L. *J. Chem. Phys.* **1993**, *98*, 10089–10092.

[7] Essmann, U.; Perera, L.; Berkowitz, M. L.; Darden, T.; Lee, H.; Pedersen, L. G. *J. Chem. Phys.* **1995**, *103*, 8577–8593.

[8] Martínez, L.; Andrade, R.; Birgin, E. G.; Martínez, J. M. *J. Comput. Chem.* **2009**, *30*, 2157–2164.

[9] Hess, B.; Bekker, H.; Berendsen, H. J. C.; Fraaije, J. G. E. M. *J. Comput. Chem.* **1997**, *18*, 1463–1472.

[10] Abraham, M. J.; Murtola, T.; Schulz, R.; Páll, S.; Smith, J. C.; Hess, B.; Lindahl, E. *SoftwareX* **2015**, *1-2*, 19 – 25.

[11] Becke, A. D. *J. Chem. Phys.* **1993**, *98*, 1372–1377.

[12] Lee, C.; Yang, W.; Parr, R. G. *Phys Rev B* **1988**, *37*, 785–789.

[13] Frisch, M. J.; Trucks, G. W.; Schlegel, H. B.; Scuseria, G. E.; Robb, M. A.; Cheeseman, J. R.; Scalmani, G.; Barone, V.; Mennucci, B.; Petersson, G. A.; Nakatsuji, H.; Caricato, M.; Li, X.; Hratchian, H. P.; Izmaylov, A. F.; Bloino, J.; Zheng, G.; Sonnenberg, J. L.; Hada, M.; Ehara, M.; Toyota, K.; Fukuda, R.; Hasegawa, J.; Ishida, M.; Nakajima, T.; Honda, Y.; Kitao, O.; Nakai, H.; Vreven, T.; J. A. Montgomery, J.; Peralta, J. E.; Ogliaro, F.; Bearpark, M.; Heyd, J. J.; Brothers, E.; Kudin, K. N.; Staroverov, V. N.; Kobayashi, R.; Normand, J.; Raghavachari, K.; Rendell, A.; Burant, J. C.; Iyengar, S. S.; Tomasi, J.; Cossi, M.; Rega, N.; Millam, J. M.; Klene, M.; Knox, J. E.; Cross, J. B.; Bakken, V.; Adamo, C.; Jaramillo, J.; Gomperts, R.; Stratmann, R. E.; Yazyev, O.; Austin, A. J.; Cammi, R.; Pomelli, C.; Ochterski, J. W.; Martin, R. L.; Morokuma, K.; Zakrzewski, V. G.; Voth, G. A.; Salvador, P.; Dannenberg, J. J.; Dapprich, S.; Daniels, A. D.; Farkas, ; Foresman, J. B.; Ortiz, J. V.; Cioslowski, J.; Fox, D. J. Gaussian 09, Revision A.02. 2009.

[14] Dudev, T.; Lim, C. *J Phys Chem A* **1999**, *103*, 8093–8100.

[15] Joly, Y. *Phys Rev B* **2001**, *63*, 125120.

[16] Bunău, O.; Joly, Y. *J. Phys: Condes. Matter* **2009**, *21*, 345501.

[17] Guda, S. A.; Guda, A. A.; Soldatov, M. A.; Lomachenko, K. A.; Bugaev, A. L.; Lamberti, C.; Gawelda, W.; Bressler, C.; Smolentsev, G.; Soldatov, A. V.; Joly, Y. *J. Chem. Th. Comp.* **2015**, *11*, 4512–4521.

[18] Joly, Y.; Bunău, O.; Lorenzo, J. E.; Galéra, R. M.; Grenier, S.; Thompson, B. *Journal of Physics: Conference Series* **2009**, *190*, 012007.

[19] Rankine, C. D.; Madkhali, M. M. M.; Penfold, T. J. *J Phys Chem A* **2020**, *124*, 4263–4270.
